# Mechanism of Iron-Dependent Repressor (IdeR) Activation and DNA Binding: A Molecular Dynamics and Protein Structure Network Study

**DOI:** 10.1371/journal.pcbi.1004500

**Published:** 2015-12-23

**Authors:** Soma Ghosh, Nagasuma Chandra, Saraswathi Vishveshwara

**Affiliations:** 1 Molecular Biophysics Unit, Indian Institute of Science, Bangalore, Karnataka, India; 2 I.I.Sc. Mathematics Initiative, Indian Institute of Science, Bangalore, Karnataka, India; 3 Department of Biochemistry, Indian Institute of Science, Bangalore, Karnataka, India; Baltimore, UNITED STATES

## Abstract

Metalloproteins form a major class of enzymes in the living system that are involved in crucial biological functions such as catalysis, redox reactions and as ‘switches’ in signal transductions. Iron dependent repressor (IdeR) is a metal-sensing transcription factor that regulates free iron concentration in *Mycobacterium tuberculosis*. IdeR is also known to promote bacterial virulence, making it an important target in the field of therapeutics. Mechanistic details of how iron ions modulate IdeR such that it dimerizes and binds to DNA is not understood clearly. In this study, we have performed molecular dynamic simulations and integrated it with protein structure networks to study the influence of iron on IdeR structure and function. A significant structural variation between the metallated and the non-metallated system is observed. Our simulations clearly indicate the importance of iron in stabilizing its monomeric subunit, which in turn promotes dimerization. However, the most striking results are obtained from the simulations of IdeR-DNA complex in the absence of metals, where at the end of 100ns simulations, the protein subunits are seen to rapidly dissociate away from the DNA, thereby forming an excellent resource to investigate the mechanism of DNA binding. We have also investigated the role of iron as an allosteric regulator of IdeR that positively induces IdeR-DNA complex formation. Based on this study, a mechanistic model of IdeR activation and DNA binding has been proposed.

## Author Summary

The importance of iron for tuberculosis infection and criticality of IdeR as an iron regulator in *Mycobacterium tuberculosis* have now been well-established. Although crystal structures of the protein in different forms are available, its dynamical properties have not been explored before. Macromolecules possess an inherent dynamicity that enables them to perform a myriad of biological functions. Understanding these dynamical properties provide interesting mechanical insights about the workings of a protein. Hence, in this study we have applied molecular dynamics integrated with protein structure networks to explore the iron sensing capacity of IdeR at atomistic levels. Further, the study was expanded to investigate the mechanism of IdeR-DNA binding. Based on the ‘population shift’ model of allostery, we identify iron as an allosteric effector of IdeR. Overall, the study not only provides an insightful representation of IdeR-DNA interactions but also anchors discussion about protein-DNA interactions in general.

## Introduction

Tuberculosis (TB) remains the largest killer amongst infectious diseases globally and is caused by the pathogen *Mycobacterium tuberculosis* (*M*.*tb*). Based on the latest WHO report [1], in the year 2013, an estimated 9 million people acquired the disease and nearly 1.5 million succumbed to the disease. *M*.*tb* is a slow-growing, acid-fast bacillus that can be transmitted via the aerosol route from one infected person to another. The global burden of TB has increased further with the advent of the drug resistant strains.

Various clinical studies have indicated the importance of iron during TB infection. Pulmonary tuberculosis patients are often found to be anemic [2], and iron supplementation is seen to promote active TB [3]. Extraction of iron from the host cell is considered to be one of the crucial factors, required for *M*.*tb* virulence [4]. Iron is also equally important for the functioning of diverse processes such as oxygen transport, detoxification and DNA synthesis in the host system [5]. Hence, a stiff competition exist between the host and the pathogen for the extraction of iron, which in turn determines the outcome of infection [6,7]. On the other hand, excess of free iron can also be toxic for the organisms as free iron can undergo Fenton reaction and produce harmful oxygen radicals. This necessitates the need for fine regulation of iron concentration in both the systems. In the mycobacterial cell, iron concentration is regulated by an iron responsive transcription factor, IdeR (Iron-dependent Repressor) [8]. IdeR is a well-characterized protein, with crystal structures available at various levels of accuracy and resolution [9,10].

**Fig 1:**
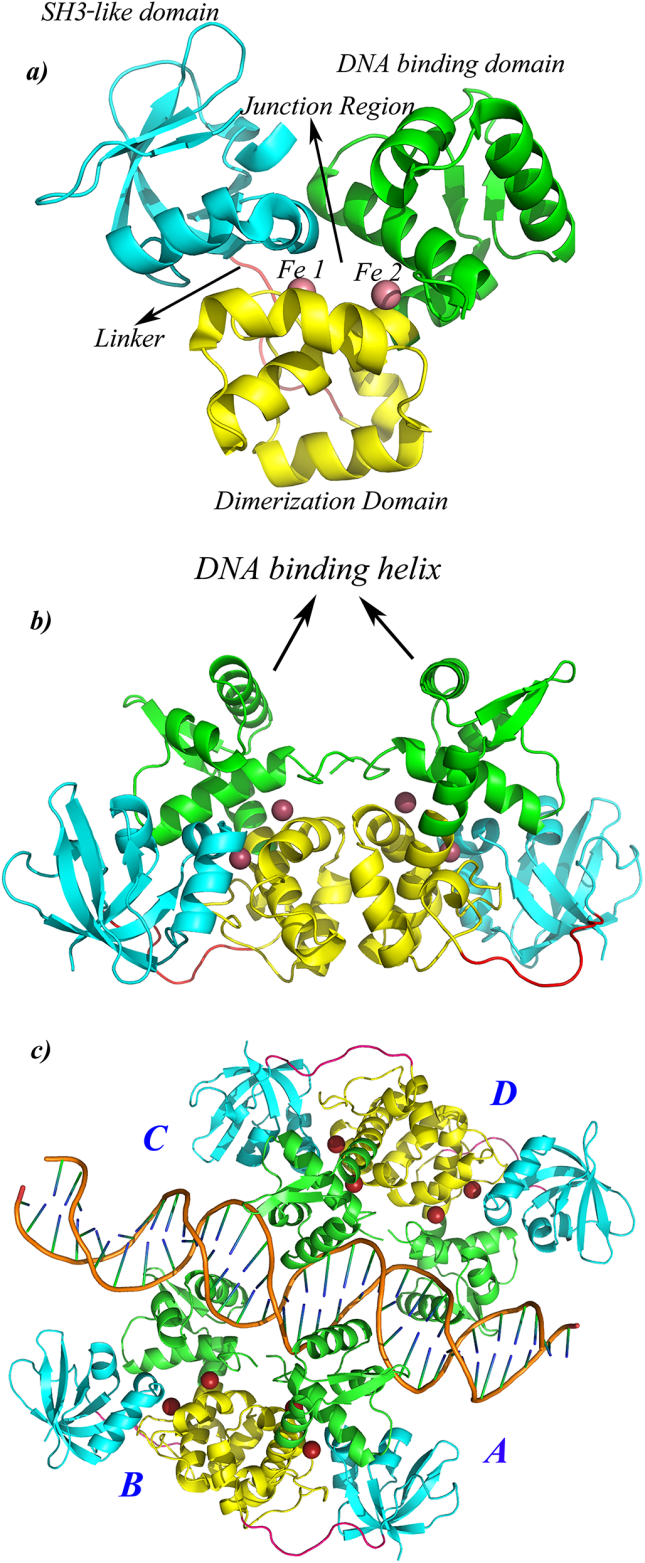
Structural details of IdeR. Cartoon representation of a) IdeR monomer that consist of three domains, DNA binding domain [DBD, green], dimerization domain [DD, yellow], the SH3-like domain [SH3, cyan] and a 10 residue long linker region [shown in pink]. Each monomer binds to two metals ions, shown in red spheres. The cavity formed at the centre of the monomer is named as the ‘Junction region’ in this study b) IdeR dimer that binds to DNA via the DNA binding helices [DBH], c) IdeR functional unit. Two IdeR dimers bind to opposite sides of DNA to form a ‘dimer-of-dimer’ complex. The four monomeric subunits are labeled as A, B, C and D.

The IdeR monomer is a 230 residue protein (Fig 1(A)) that can be divided into three domains, the DNA binding domain (DBD) from residues 1 to 74, dimerization domain (DD) formed by residues 75 to 140 and the SH3-like domain (SH3), involving residues 151 to 230. While DBD and DD domains are involved in DNA binding and dimerization, respectively, the exact function of the SH3-like domain is still debatable. While some studies suggest it to be important for monomer stability, others have related it to DNA binding[11]. Residues 141 to 150 represent the linker region that connects the SH3-like domain to the dimerization domain. Two monomeric subunits interact with each other to form a dimer that binds to DNA, mainly via the DNA binding helices (DBH) as illustrated in Fig 1(B).Two IdeR dimers bind to opposite sides of a 19bp conserved DNA sequences, called the ‘iron box’[8,10]to form a dimer-of-dimer complex as shown in Fig 1(C).

Presence of two metal binding sites in IdeR monomer bestows it with its iron sensing capacity. The first metal binding site (MS1), also known as the ancillary site forms a distorted bipyramidal geometry with Fe^+2^ using side chain atoms of five residues (His79, Glu83, His98, Glu172, Gln175), while the second metal binding site (MS2) or the primary site forms an octahedral geometry with Fe^+2^ involving four residues (Met10, Cys102, Glu105, His106) and a water molecule. A detailed representation of the two metal binding sites is shown in S1 Fig. In solution, IdeR exists as monomer-dimer equilibrium, with iron binding shifting the equilibrium towards dimer formation [11]. Based on this observation, a strong coupling between metal binding and dimerization has been suggested.

In this study, we have investigated the influence of iron on the structure and function of IdeR, which in turn triggers a cascade of events responsible for maintaining iron concentrations in the bacterial cell. Our simulations clearly indicate the importance of iron for structural stability and for preparing the dimer for DNA binding. However, the most striking results are obtained from the simulation studies of IdeR-DNA complex in the absence of iron, where the protein-DNA complex shows signatures of dissociation, whereas, the same complex remains stable in the presence of iron. We have also investigated the possible allosteric nature of IdeR-DNA binding, using the population shift model of allostery. Finally, based on the experimental details available and observations from the simulations, we propose a model of IdeR activation and DNA binding. The model not only provides a general understanding of the mechanisms of protein-DNA interactions, it also provides a framework for testing novel drug designing strategies for TB.

## Results

Simulations were performed for 100 ns on 7 different systems that represented the different forms of IdeR. These include the monomeric forms (M, M1, M2), the dimeric forms (D-nm and D-m) and the functional unit or the IdeR-DNA complex (FU-nm and FU-m), each with or without iron ions bound to them. Details pertaining to the structures are listed in Table 1. The influence of iron on IdeR was clearly evident in all the structural forms. Here, we mainly focus on the functional unit of the protein. However, a brief discussion of the important results pertaining to the monomeric and dimeric forms of IdeR is also presented and details are given in S1 Text and S2 Text.

**Table 1:** List of systems selected for simulation studies.

*System Nomenclature*	*Oligomeric state*	*Iron @ MS1*	*Iron @ MS2*	*DNA*
M	Monomer	No	No	No
M1	Monomer	Yes	No	No
M2	Monomer	Yes	Yes	No
D-nm	Dimer	No	No	No
D-m	Dimer	Yes	Yes	No
FU-nm	Dimer-of-dimer	No	No	Yes
FU-m	Dimer-of-dimer	Yes	Yes	Yes

### IdeR monomer exhibits conformational flexibility: The ‘open’ and ‘close’ conformations

The C^α^ RMSD profiles and the residue wise fluctuation plots (S2 Fig) obtained from the simulations of the three monomeric systems clearly suggested higher fluctuations in the non-metallated monomeric system. This was also confirmed by mapping the direction and magnitude of the first principal component on the protein (S3 Fig). Porcupine plots of the protein (using PC1) also indicated that the SH3 domain and the DBD move in opposite directions in the absence of iron and towards each other upon iron binding. Additionally, the magnitude of movement is much higher in the apo form (M) as compared to the metal bound structure (M2). The two opposing domain motions yield two conformations, the ‘open’ conformation, where the domains move away from each other and the ‘close’ conformation, where the domains move towards each other. Interdomain angles have been used to quantitatively characterize the domain motions in the three monomeric systems (presented in S1 Text), and the detailed structural features of the two conformations have been identified and discussed.

**Fig 2:**
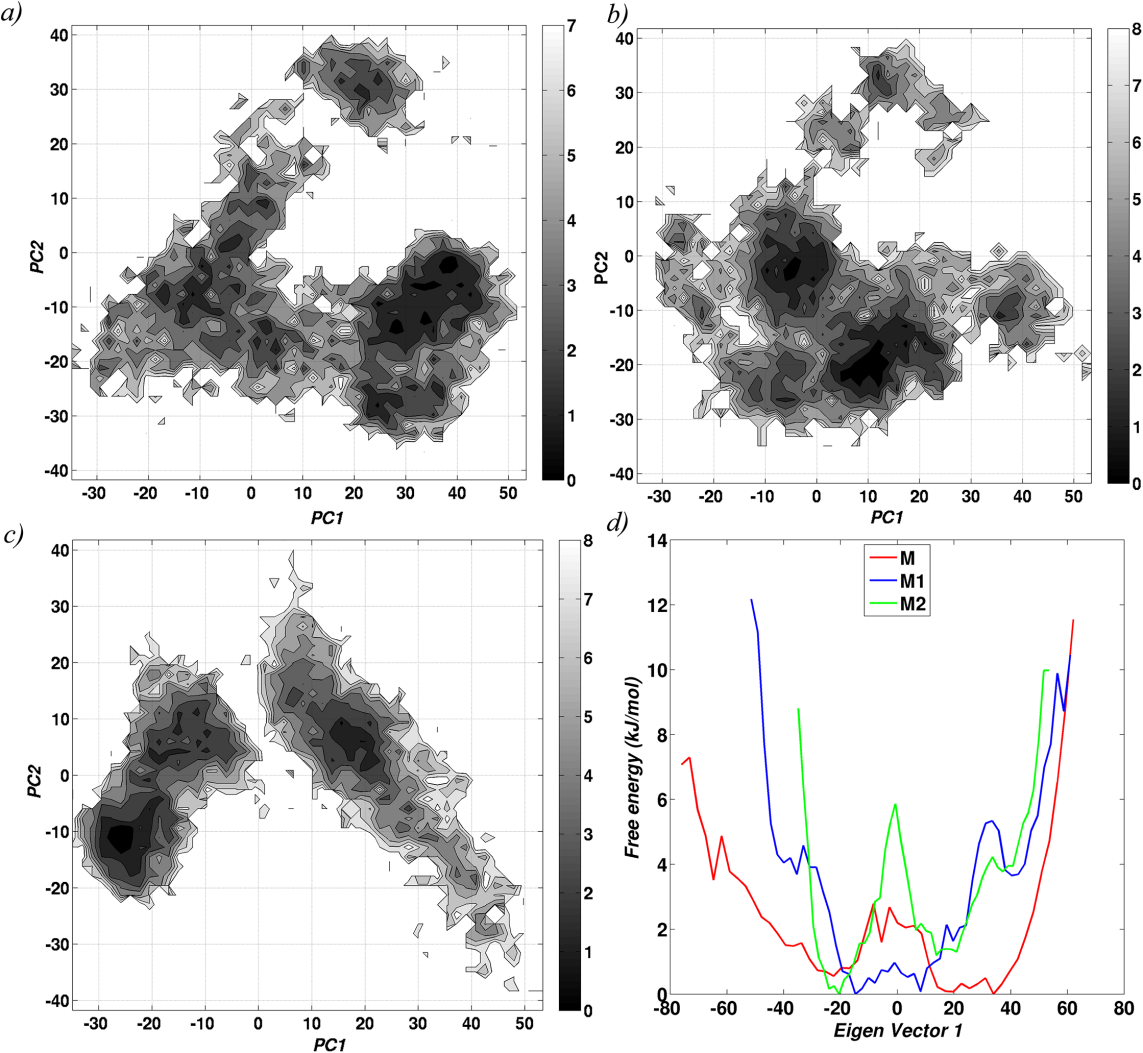
Conformational flexibility of the monomeric systems. Free energy landscape based on the first two eigen vectors from the simulation trajectories of monomeric units are shown for a) M (without metal ion), b) M1 (bound to one Fe+2 ion) and c) M2 (bound to two Fe+2 ions).d) Free energy landscape based on the first eigen vector for all the three system.

The energetics involved in conformational switching in the three monomeric systems was also explored using the concept of statistical free energy landscape. The conformations taken up by the three monomeric systems were projected on the “essential plane” to obtain a population distribution profile and the energetics (Fig 2). The broad spectrum of the landscape in the apo form (M), confirms the presence of higher conformational flexibility, accompanied by a relatively easy transition between the different conformations. Such conformational transitions become increasingly infeasible when iron is bound to the system (M2), as indicated by the two minima in the energy landscape. Interestingly, the energy landscape of one iron bound monomer (M1) behaves as an intermediate of the M and M2 forms. The above observations are much more evident from the one dimensional free energy landscape plot (Fig 2(D)), which was plotted using only the top principal component.

**Fig 3:**
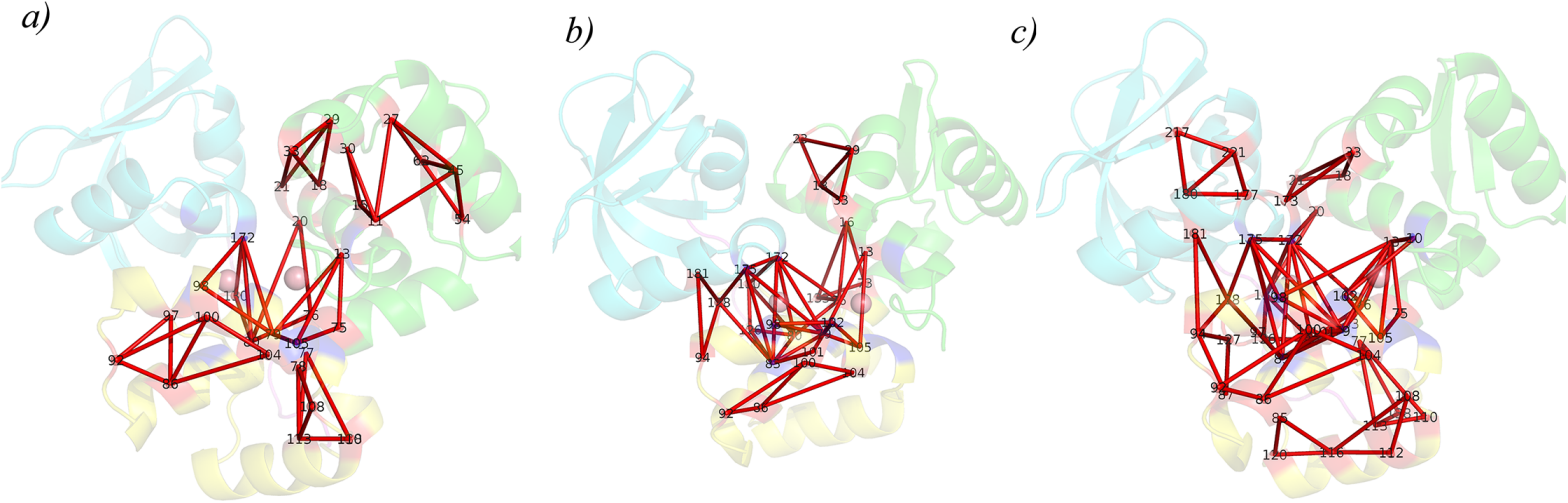
The structural readjustments as a result of iron binding were also evident from protein structure networks. Dynamically stable community formation in the PSNs of the three monomeric systems, a) M, b) M1 and c) M2 is illustrated. Communities are indicated by red triangles and calculated using the program PSN-Ensemble.

Structural modifications in IdeR as a result of iron binding was also assessed using protein structure networks. Fig 3 shows the overall network connectivity in terms of community structures and distinctly highlights the influence of iron on domain motion and conformational preference. In the apo form (M), the inter-residue connections, as determined by community formation were found to be sparse and mainly involved residues from the DNA binding domain (DBD) and the dimerization domain (DD). Residue, Glu172, which is a metal binding residue (MBR) formed the only link between SH3 domain and the other two domains, thus keeping the SH3 domain flexible. Such flexibility is consistent with the preference for the ‘open’ conformation in the apo form. We also noted the formation of a well-connected component near the DBD in the apo form. Upon iron binding (M1, M2), these patterns changed significantly, and resulted in the formation of a bigger and stronger community at the junction region. This involved residues from all the three domains, clearly indicating an increase in interdomain interactions and hence, a preference for the ‘close’ conformation. Intriguingly, however unlike the other regions of the protein, the DBD attains flexibility in M1 and M2. This increased flexibility of the DBD upon iron binding could have an implication in DNA binding and has been discussed in detail in a later section.

### Stability of N-terminal residues in IdeR dimers

**Fig 4:**
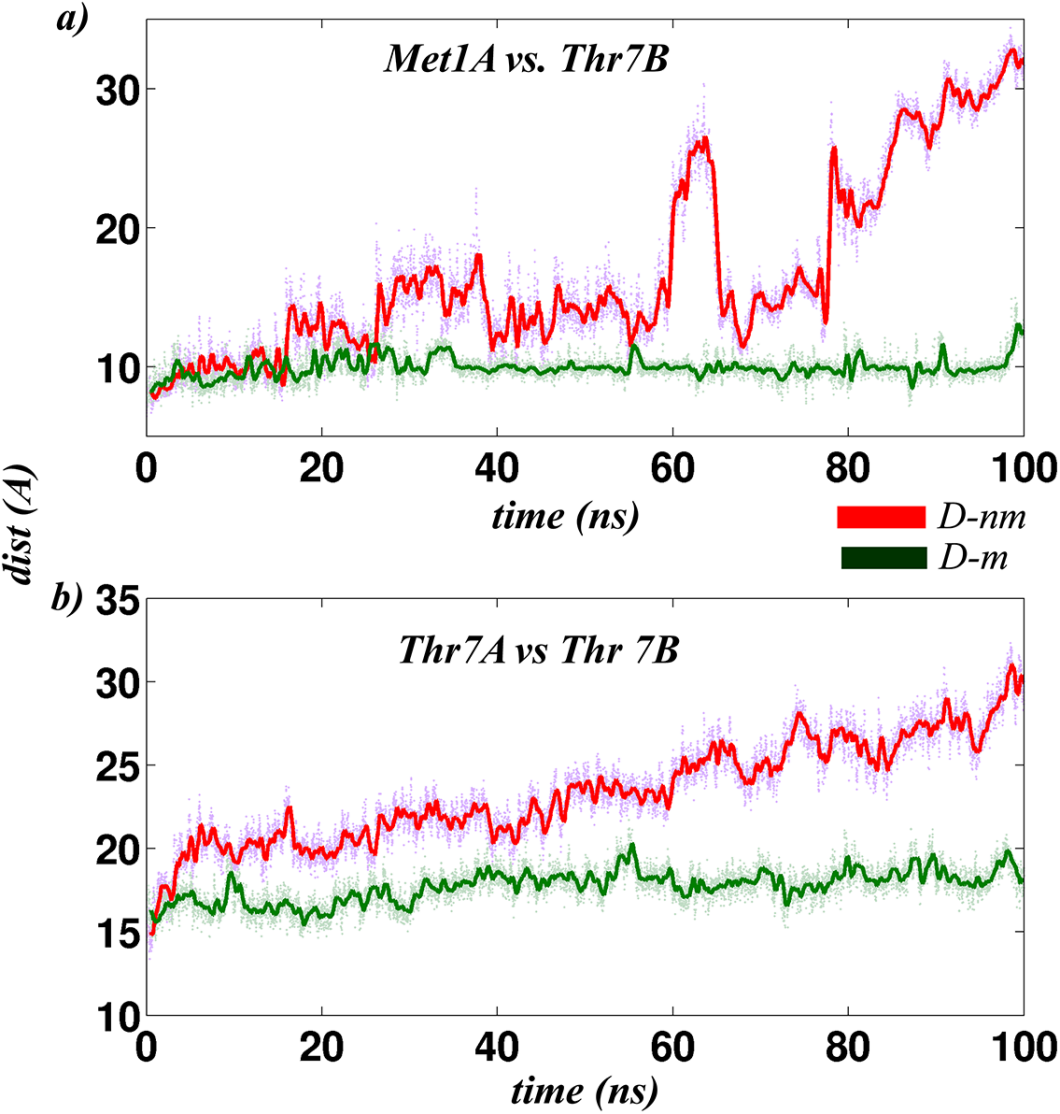
Distance profile of the N-terminal residues between the two chains as a function of simulation time. N-terminal stability between the two dimeric systems (with and without metal ion) is quantified by measuring distances between a) Thr7A-Met1B and b) Thr7A-Thr7B as a function of time over the entire period of simulation (100 ns).

In the next step, IdeR dimers were simulated in the presence (D-m) and absence of metal ions (D-nm) to investigate the influence of metal ions on the stability of N-terminal residues. This was analyzed using interface clusters (see S2 Text) and distance profiles of specific pair of N-terminal residues (Met1 and Thr7) from the two chains (Fig 4). A striking contrast between D-m and D-nm was seen in these distance plots, with D-m exhibiting lower distance values than D-nm. Stability of the N-terminal residues in D-m strongly implicates its role in protein functioning. A careful observation of the dimer structures highlighted the importance of a specific orientation of the DBHs. DBHs are known to majorly interact with DNA, and therefore fixing the relative position of DBH is crucial for DNA binding. We postulated that stability of the N-terminal residues and interaction between the N-terminal residues from both chains was important for reorienting the DBHs in a particular position, such that it can exactly fit into the DNA grooves. Stability of the N-terminal residues has also been indicated in experimental studies [11] and was confirmed by our simulation studies. The atomic details of how iron stabilizes the N-terminal residues are discussed in S2 Text and a significant result from that is discussed here.

Comparison of the hydrogen bonds in the two dimeric systems showed that Met10, an N-terminal residue formed extensive connections with the N-terminal residues as well as the metal ions. Taken together, this reduced the flexibility of the N-terminal residues in the metallated system. Mutation of Met10 is also known to abolish protein functioning in the IdeR homologous protein, DtxR[12]. In the case of IdeR, our simulation studies suggested the importance of Met10 for stabilizing the N-terminal residues, which in turn helped in fixing the position of DBHs that seems to be crucial for DNA binding.

Overall, simulations of the protein subunit in its monomeric and dimeric forms clearly demonstrated the role of iron in structure stabilization. It was also noted that while iron induced structural stabilization between the three domains, the DNA binding domain increased its flexibility upon iron binding. This may have implication in DNA binding. Additionally, stability of the N-terminal residues could also play a crucial role in DNA binding. It seems that IdeR is predisposed for DNA binding even in the absence of DNA, but surely in the presence of metal ions. In the next section, we further discuss these aspects based on the simulations of the protein in complex with DNA, in the presence and absence of iron.

### Influence of iron on IdeR-DNA complex, the functional unit

The DNA and metal ion being oppositely charged, it is evident that the metal ion brings stability to IdeR-DNA complex. However, the question to be addressed was regarding the details of the specific and the non-specific interactions that lead to the stability of the complex. Such a study would provide the residue details crucial for specific binding and also provide insights into the mechanism of DNA binding. To investigate this, we performed 100 ns simulations on the IdeR dimers bound to DNA in the presence (FU-m) and absence (FU-nm) of iron. The results were analyzed to bring out the differences in the nature of IdeR-DNA binding from these two simulations and to affirm the conjectures proposed above.

#### Global structural deviations

The stability of the complex was assessed by calculating the C^α^ RMSD of the protein subunits (S4(A) Fig) with reference to their final equilibrated structures generated at the end of 120ps simulation (Table B in S3 Text), henceforth denoted as the ‘reference’ structure. A significant deviation of ~ 11 Å was observed for FU-nm as compared to FU-m, which was within 6Å. Interestingly, all-atom RMSD for the DNA subunit (S4(B) Fig) alone did not show such a drastic variation, with RMSD values being below 5Å in both cases. Absence of a large structural deviation in the DNA component indicated that the large structural deviation observed for the IdeR-DNA complex (FU/FU-nm) was a result of structural deviation in the protein subunit and not the DNA subunit.

**Fig 5:**
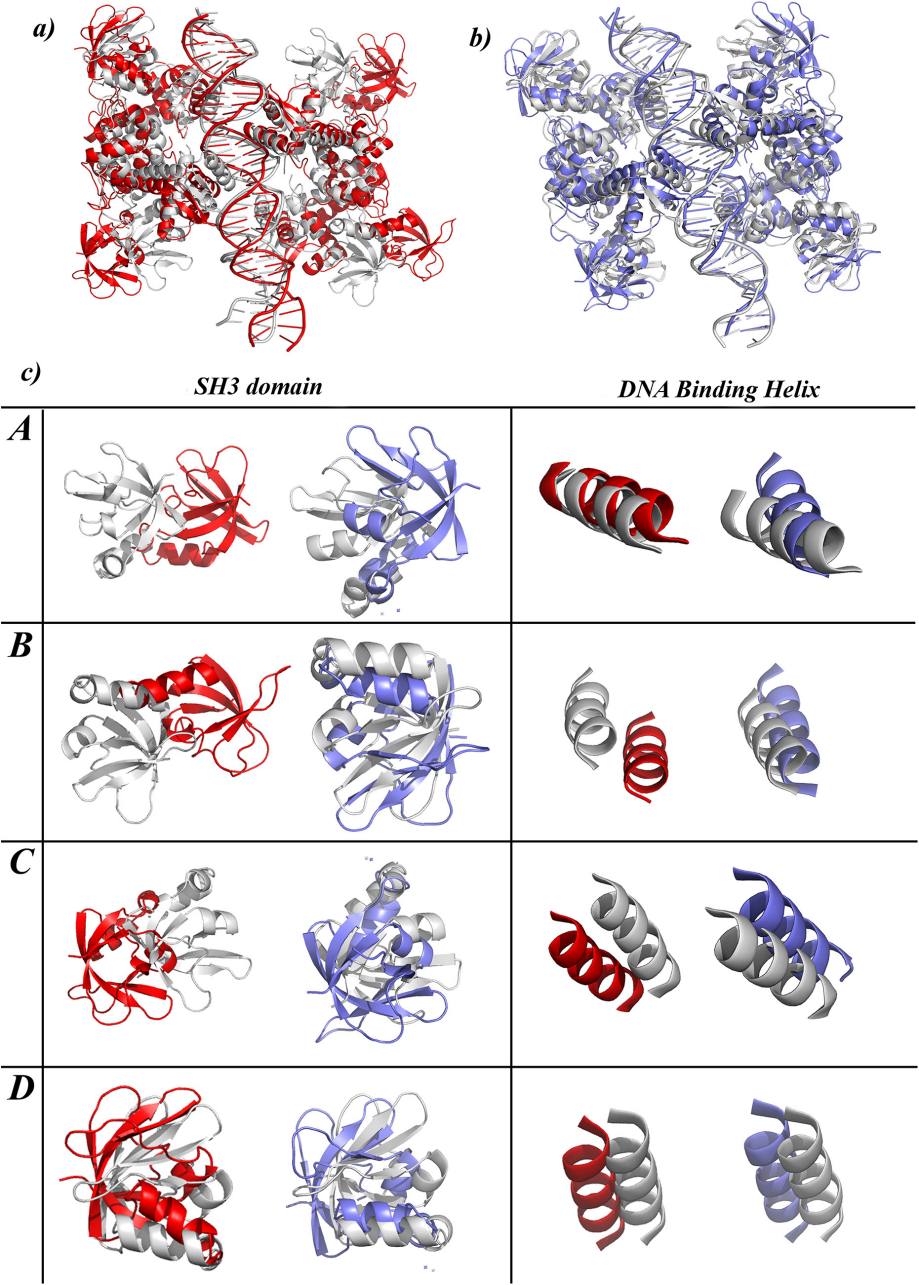
Structural superposition of the reference and final structure of a) FU-nm and b) FU-m are shown as cartoon representation. In both cases the reference structures are shown in gray while the final structure obtained at the end of 100 ns simulations are shown in red for FU-nm and blue for FU-m. c) Panel highlights two specific regions in the structure that shows maximum deviation in the non-metallated case. The first column shows variation in the SH3 domain, while the second column represents variation in the DNA binding helix [DBH]. Each row in the panel represents the four monomeric subunits.

The structural deviations in the protein subunits were further analyzed by superposing the ‘reference’ structure and the final structure and identifying the regions of structural variations (Fig 5A and 5B). A close inspection revealed independent deviations in the four monomeric units that arose at two specific regions, the SH3 domain and the DNA binding helix (DBH). Movement of SH3 domain was related to the opening and closing of the domains which was also observed in the monomeric simulations. On the other hand, deviations observed in DBH related to a rigid body movement, wherein the complete monomer moved away from the DNA. The details of these deviations are clear from Fig 5(C). Each box in the figure illustrates the differences observed in each monomeric subunit between the metallated and the non-metallated system.

**Fig 6:**
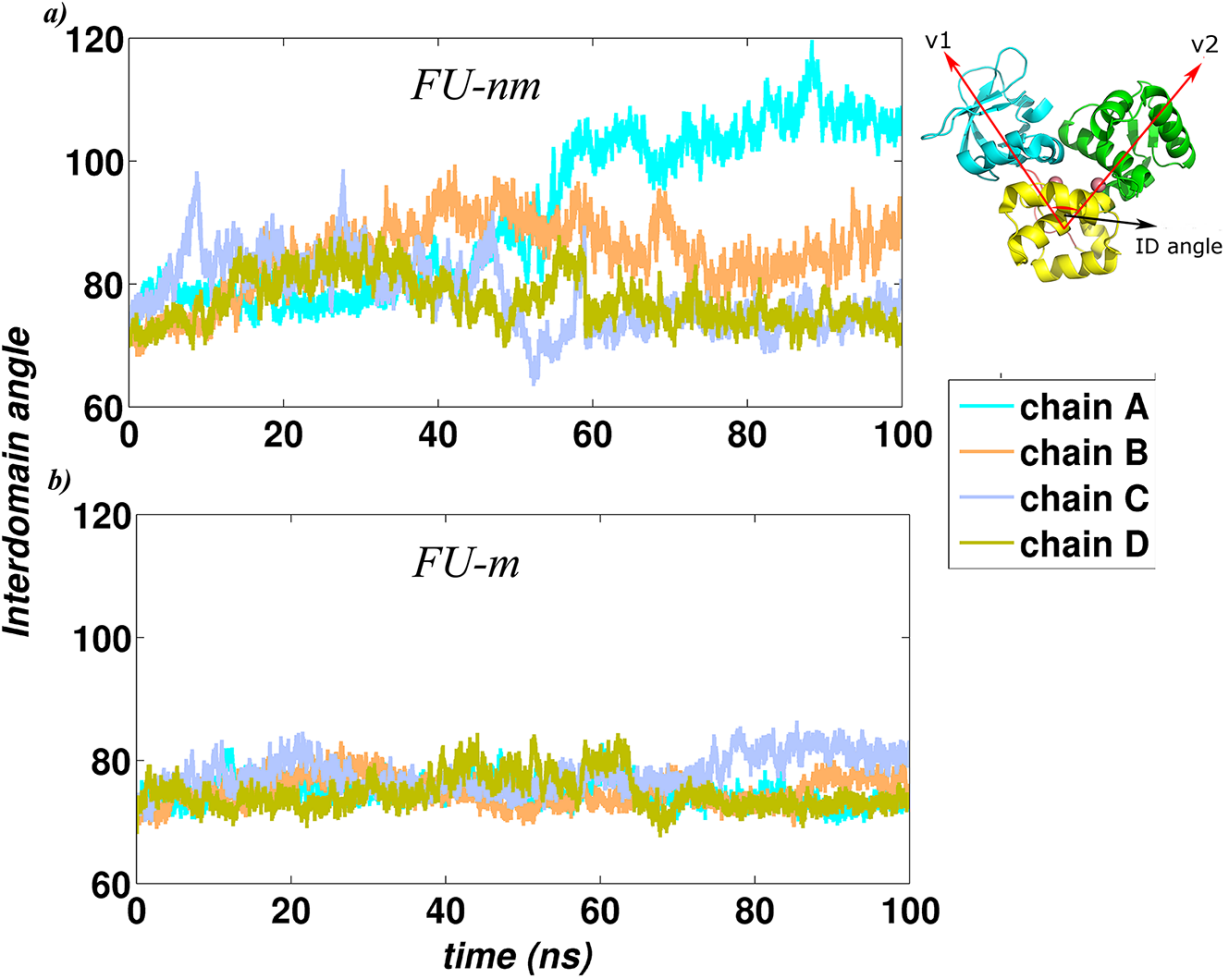
The trajectories of the interdomain angle (the angle between the centers of mass of the three domains, as shown in the inset) for all the subunits in the two functional units, a) FU-nm and b) FU-m. Different colours represent different chains in the system.

The deviations of the SH3 domain were also quantified by calculating the interdomain angle of all the monomeric units that formed the complex, similar to that calculated for the monomeric simulations (Fig A in S1 Text). As can be seen from Fig 6, the interdomain angle exhibited higher fluctuation in FU-nm as compared to FU-m, indicating a continuous transition between the ‘open’ and the ‘close’ conformations in the monomeric subunits of the non-metallated system. Amongst the four monomeric subunits in FU-nm, two were seen to take up the ‘close’ form while the other two remained in the ‘open’ conformation, hinting into an asymmetric nature of protein-DNA dissociation. On the contrary, the monomeric subunits in the metallated system were quite stable with the interdomain angle lying within a very narrow range.

#### Residue level interactions

Besides the global structural modifications discussed above, IdeR-DNA interactions were also estimated based on three parameters, a) number of hydrogen bonds, b) network of non-covalent interactions and c) MMPBSA based energy calculations. These energetic features of the IdeR-DNA complex, in the absence and presence of iron are discussed in this section.

Hydrogen bonds between DNA and protein: Hydrogen bonds that were present in at least 50% of the snapshots were considered for this analysis. Hydrogen bonds formed between protein residues and DNA for each chain of all the systems are summarized in Table C in S3 Text. Extensive loss of hydrogen bonds was seen in FU-nm as compared to FU-m and correlated well with rigid body motions observed for the individual monomeric subunits. These hydrogen bonds re-emphasized on the asymmetric nature of dissociation between IdeR and DNA. Similar asymmetric behavior has also been indicated by experimental studies performed on protein-DNA binding. These studies have suggested “hemispecific” binding in DNA[13], wherein one segment of the protein interacts strongly with the DNA as compared to the other. In fact, kinetic studies on IdeR [11] have also suggested the possibility of such hemispecific behaviour, wherein, DBH from one of the chain initially interacts with the cognate DNA and anchors itself, followed by the binding of the other chain. Our simulations have been able to show that similar asymmetry is also observed in the process of dissociation, which happens in the absence of metals.

We also observed higher contribution of N-terminal residues in hydrogen bonding in FU-m, confirming the role of N-terminal residues for DNA binding as also suggested earlier. This was speculated by us on the basis of observations from the dimer simulations. Amongst the different residues that form hydrogen bonds with DNA, special attention should be given to Gln43. This is the only residue that forms hydrogen bond with the nucleobases, thus indicating higher specificity. Interaction of protein residues with DNA bases are considered to be highly specific in nature as opposed to the non-specific electrostatic interactions with the negatively charged phosphate backbone of DNA. These interactions play a pivotal role in identifying the DNA binding sites and in many cases initiate the process of DNA binding. In the IdeR-DNA complex, Gln43 was seen to interact with guanine or cytosine from DNA, however this interaction was not present in all the monomeric subunits of the protein. Probing further, it was noted that the orientation of Gln43 determined the formation of hydrogen bonds (S5 Fig). For instance, in the case of FU-m, Gln43 from chain B and D were located below and/or above the DNA and provided easy access to the nucleobases to form hydrogen bonds, as compared to chain A and C where Gln43 was present along the DNA backbone. It is interesting to note, how minor differences such as this can impact interactions between protein and DNA and could in fact form the basis for “hemispecific” binding. Such specific interactions were not observed in FU-nm, highlighting the importance of iron for strong IdeR-DNA interactions.

Protein-DNA interface clusters: Besides hydrogen bonding, non-covalent interactions also played an important role in stabilizing the protein-DNA complex. These interactions were investigated by calculating interface clusters from the protein-DNA bipartite network (PDG). A detailed list of residues involved in cluster formation is provided in Table D in S3 Text. Overall observations from network analysis correlated with the hydrogen bond analysis. Involvement of N-terminal residues towards cluster formation drastically increased in the metallated system. On the other hand, in the non-metallated system only a few residues were seen to contribute towards complex formation and a decrease in the participation of the N-terminal residues as compared to the metallated systems was also observed. The DNA phosphate backbone was found to interact with a stream of residues along the stretch of DNA-protein interface that helped in stabilizing the overall structure.

Non-covalent interactions of protein residues with the nucleobases indicated that all subunits formed a minimum of one cluster that involved nucleobases, with the exception of chain B and C of FU-nm. In the case of hydrogen bonds, interaction with nucleobases was limited to a single residue (Gln43) as compared to the non-bonded interactions. Furthermore, it was also noted that the protein residues in these clusters mostly belonged to the DBHs, indicating the importance of this segment in imparting specificity. From the above observations, it could be concluded that a strong interaction of IdeR with DNA is only possible when residues from different pockets of DBD (DBH, 2^nd^ helix and N-terminal) contribute together towards complex formation. This is exemplified in chain D of FU-m, where interface clusters involves residues from all the three sites (Table D in S3 Text).

Residue–residue interaction energy at IdeR-DNA interface: To obtain an estimate of the strength of protein–DNA interactions, MMPBSA analysis was carried out to calculate the free energy of complex formation. This was found to be lower for FU-m (-260 kcal/mol) as compared to its non-metallated counterpart which was equal to -169 kcal/mol. A difference of ~100 kcal/mol between the two complexes provided clear evidence on the importance of metal in DNA binding.

Additionally, we also calculated the energetic contribution towards the free energy of complex formation for different pairs of residues. Table E in S3 Text lists the top 20 DNA-protein residue pairs that contribute maximally towards complex formation in FU-nm and FU-m. Each pair in the table is annotated according to the location of the protein residue. As expected, maximum contribution towards complex formation was seen from residues belonging to DBH, 2^nd^ helix or the N-terminal region. Specifically, Arg60 from the 2^nd^ helix contributed significantly towards complex formation. Arg60 was also seen to form hydrogen bonds and interface clusters.

#### Role of N-terminal residues in DNA binding

The role of N-terminal residues was from the dimer simulations and from the residue level analysis of the IdeR-DNA complex. Here, we further probed into this finding by analyzing the long range communications between the metal binding residues and DNA in terms of hydrogen bonds.

A hydrogen bond network, involving protein and DNA residues was generated. The nodes were connected if a hydrogen bond with occupancy ≥ 30% existed between the nodes. The network was used to calculate shortest paths between all possible pair of nodes using the Floyd-Warshall algorithm [14]. Shortest paths between two nodes are indicative of the cascade of hydrogen bonds that connects distant segments of the complex. As opposed to direct hydrogen bond interactions analyzed in the previous section, the focus in this section shifted to the long range influences that helps stabilize the protein-DNA complex. More specifically, the communications between metal binding residues [MBR] and DNA via the N-terminal residues were probed.

The hydrogen bond network generated, contained 986 nodes and 740 edges for FU-nm and 774 edges for FU-m. A total of 2036 all-pair shortest paths were obtained for FU-nm and 3196 for its metallated counterpart, strongly highlighting the connectivity in the metallated system. More significantly, about 20.21% of the shortest paths in FU-m involved at least one N-terminal residue as compared to 6.09% for FU-nm. The increase in the involvement of N-terminal residues for the overall communication within the protein-DNA complex is evident from the percentage values.

Further, to evaluate the role of N-terminal residues in DNA binding, shortest paths between protein and DNA residues were extracted from the total list of all possible shortest paths. 152 such shortest paths were obtained for FU-nm as opposed to 586 in FU-m. Analysis and comparison of the paths from the networks derived from the two systems indicated that none transited via N-terminal residues in the case of FU-nm as compared to 240 paths in FU-m. In other words, the N-terminal residues played negligible role towards IdeR-DNA interactions (at the level of hydrogen bonds) in the absence of iron. Dwelling deeper, 14 of the 240 shortest paths obtained from the network of FU-m, also involved metal binding residues. A brief summary of the different calculations performed using the hydrogen bond network is shown in S6(A) Fig, while the hydrogen bond connections representing the 14 shortest paths (between protein and DNA, and transitioning from N-terminal residue and metal-binding residue) is plotted in S6(B) Fig.

Interestingly, the 14 shortest paths also indicate an asymmetric behavior of IdeR-DNA binding, with one of the dimer (Dimer 1, chain A and B) being actively involved in forming the cascade of hydrogen bond as compared to the other dimer. This is also illustrated in (S6(B) Fig). Simulations of the IdeR-DNA complex have indicated asymmetric nature of DNA binding/dissociation from multiple perspectives and can be considered as one of the probable mechanisms of IdeR-DNA interactions. This has been discussed in details in the next section.

## Discussion

IdeR is an essential protein for *Mycobacterium tuberculosis* and responsible for controlling free iron concentration inside the pathogen. It is now well established that iron plays an important role in bacterial virulence and its survival inside host macrophages [5,7,8]. In our earlier study we had built a rule based model of the host-pathogen interaction for the uptake of iron [6] upon tuberculosis infection. Analysis of the model identified IdeR as a critical component that regulates the iron extraction machinery as well as iron concentrations in the bacterial cell. This lead us to perform a molecular level study of IdeR to obtain a detailed understanding of influence of iron on IdeR as well as to study interaction between IdeR and DNA.

Simulations were performed for seven different systems, each reflecting different phases that the protein takes up to perform its function. Comparison of the metallated and the non-metallated system, clearly establishes the significant role played by iron in preparing the structure for DNA binding. The factors that regulate IdeR-DNA interactions have been analyzed using a combination of molecular dynamics simulations and protein structure networks. Investigating the dynamic properties of IdeR-DNA interactions has enabled us to study specific aspects of IdeR-DNA interactions, each of which has been discussed in details below.

### Is IdeR-DNA binding allosterically regulated?

The phenomenon of ligand binding at one site of a protein, inducing changes at a distant site to elicit physiological response, is known as allostery in general terms. Ligands that bind to the first site are termed as the ‘effector molecules’ [15]. This classical definition of allostery was coined decades ago in the ‘MWC’ model [16] or the ‘Pauling-KNF’ model [17]. Since, its inception, many studies have been performed to explain allostery in different proteins which has led to an advancement in understanding allostery from various perspective. Allostery was first described in the context of multimeric proteins where the structure generally underwent major conformational changes [16,17]. Such a phenomenological view was supported by structural studies [18,19] that mainly involved static crystal structures of the protein molecule with and without effector binding. However, experiments have shown that allostery can exist even in monomeric proteins [20]. More importantly, the conformational changes can be as large as rigid-body motions or as subtle as side-chain reorientation [21]. Encompassing such a wide spectrum of examples begs for a generalized theory to explain allostery. The advent of advanced experimental as well as computational methods, such as NMR and molecular dynamics, brought the ensemble view of conformation as the basis of this phenomenon. According to this view, the equilibrium structure of a protein is not a single structure, but it is a collection of several conformations. This can be described in terms of the population of different conformations or in terms of free-energy landscape at a more fundamental level. Thus, the definition of allostery in terms of the shift in conformational population or energy-landscape due to perturbation (by the effector molecule or change in the environmental condition) is emerging as the basic principle of allostery [21–23]. In fact the same principle has also been described to explain enzyme catalysis [24]. Such conformational changes in a protein can also directly influence protein-protein interaction and signaling networks of the cell, thereby having a larger impact on the system as a whole. These concepts have paved way to study drugs that can have indirect influences in a cellular system as a result of its allosteric influence on one of the proteins [25].

The ensemble view of allostery emphasizes on three things, a) change in conformational landscape as a function of effector binding (even the classical models, agreed on the importance of conformational changes), b) pre-existence of all the conformations, albeit with unfavourable energetics, c) environmental factors such as pH, temperature and small ligands can also act as allosteric effectors, since these factors have the capability to alter the conformational landscape of a protein in a manner that evokes function. Thus, the concept behind allostery is wildly expanded. It encompasses structural variations to changes in equilibrium populations, reflecting changes in the energy landscapes.

Here, we demonstrate the allosteric role of iron through the energy landscape model. It is to be noted that the simulations are not long enough to monitor the formation of complex between the DNA and protein. However, some of the events based on the dissociation of the subunits observed in the absence of metals can be used to understand allostery in this protein-DNA complex.

**Fig 7:**
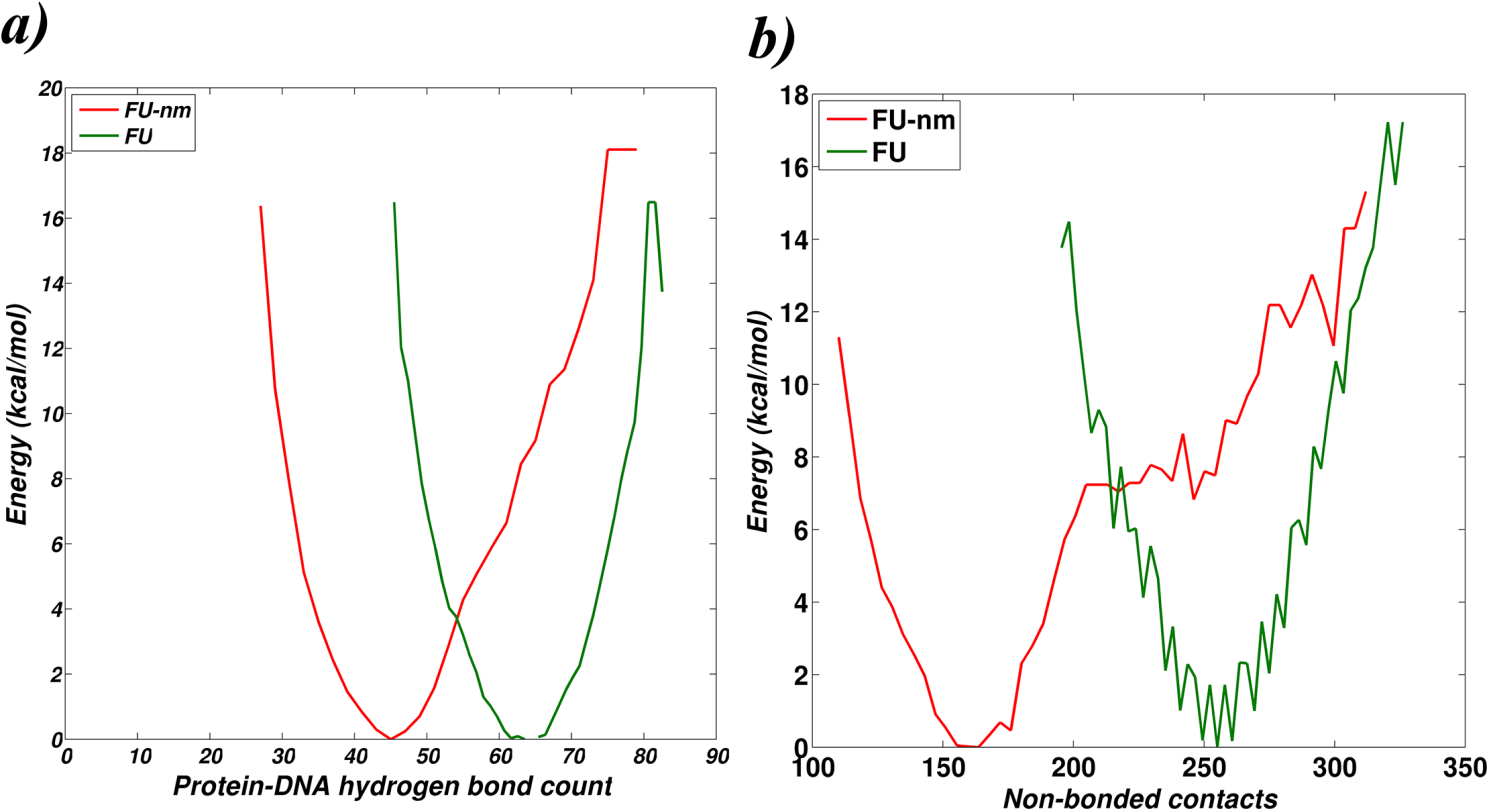
Influence of metal on the energy landscape of IdeR-DNA complex. Y-axis represents the statistical free energy evaluated with respect to a) number of hydrogen bonds between protein and DNA and b) the number of non-bonded contacts between the protein and DNA. These two parameters broadly define the extent of protein-DNA interaction. Profiles shown in red are for the non-metallated system [FU-nm], while those in green are for the metallated system [FU-m].

A shift in the statistical free energy landscape of the protein-DNA complex, plotted as a function of hydrogen bonds and non-covalent interactions, is observed upon iron binding (Fig 7), thereby indicating the role of iron in bringing out changes in the free-energy landscape. The number of protein-DNA hydrogen bonds was calculated using MolBridge [26], and non-bonded contacts were identified from the bipartite graphs between protein and DNA (PDGs). These two factors directly correlate to the stability of the complex and hence have been used for this analysis. An increase in the number of protein-DNA interaction (hydrogen bonds as well as non-bonded contacts) is observed for the metallated case as compared to the non-metallated case. Apart from the shift in energy landscape upon iron binding that stabilizes the functional complex, iron binding is also seen to induce local refolding of N-terminal regions and increase flexibility of DNA binding helices. In addition, within the protein, the iron binding site and the DNA binding site are distantly located. All the above observations enable us to predict that iron regulates IdeR-DNA interactions allosterically. Reports [27,28] have listed many examples where binding of effector ligand lead to local folding which then regulates the function.

### Mechanism of IdeR-DNA interactions

Here we provide some insights into the mechanism of association-dissociation of IdeR-DNA complex by scrutinizing the dynamics of FU-nm at residue level detail. Our simulations indicate that interaction of IdeR with DNA could be regulated either at the level of a) ‘anchor’ DBHs, wherein one of the DBH within a dimer binds strongly and regulates the binding of the other DBH or b) the dimeric unit first binding to DNA that regulates the binding of the other dimeric unit. The two mechanisms are perhaps a manifestation of the control exerted by iron towards DNA binding and are discussed below.

Providing evidence for the first model (a), we observe that over the entire simulation of FU-nm, DBH of one of the chains from both the dimeric units always remain bound to the DNA, while the other DBH moves away from the DNA (Fig 5). This two-step dissociation of the complex could also reflect the association mechanism wherein in the first step; one of the monomeric subunits initially anchors to the DNA which promotes the binding of the other subunit in the second step. This is further confirmed by measuring the DNA-DBH distance of all the chains in the two functional units studied here (S7 Fig). The behaviour of DBH-DNA distance profiles support the mechanism of an anchoring DBH that initiates the process of binding and also regulates the binding of the second DBH from the other subunit.

The second mode of interaction (b) assumes that one dimeric unit initiates the binding of the second unit and is investigated here. This has been examined based on the profiles of interdomain angles of each monomeric subunit in the complex (Fig 6) and the location of the 14 shortest paths between DNA and protein residues and involving N-terminal residues and metal binding residues, obtained from the analysis of the hydrogen bond network (S6(B) Fig). Evaluation of the interdomain angles of FU-nm suggest that monomeric subunits belonging to one of the dimers (Dimer 1) is larger as compared to the monomeric subunits of the other dimer. These monomeric subunits take up the ‘open’ conformation in the non-metallated case (FU-nm). Additionally, the 14 shortest paths (from hydrogen bond network of FU-m) suggest higher activity of one of the dimers (Dimer 1). All this suggest that the two dimers behave asymmetrically.

All the above clearly indicate the influence of iron on IdeR conformation and free energy landscape, which in turn facilitates DNA binding. Our analysis at the residue level supports the two step process of binding where DBH of one of the monomeric unit (within a dimer) acts as an anchor and facilitates the binding of the other monomeric unit. We also observe asymmetric modes of DNA binding by the two dimeric units. Asymmetry has also been observed in previous studies of tRNA synthetases [29–31] and also with the concept of allostery coupled to enzyme catalysis [32].

### Proposed model of IdeR activation and DNA binding

**Fig 8:**
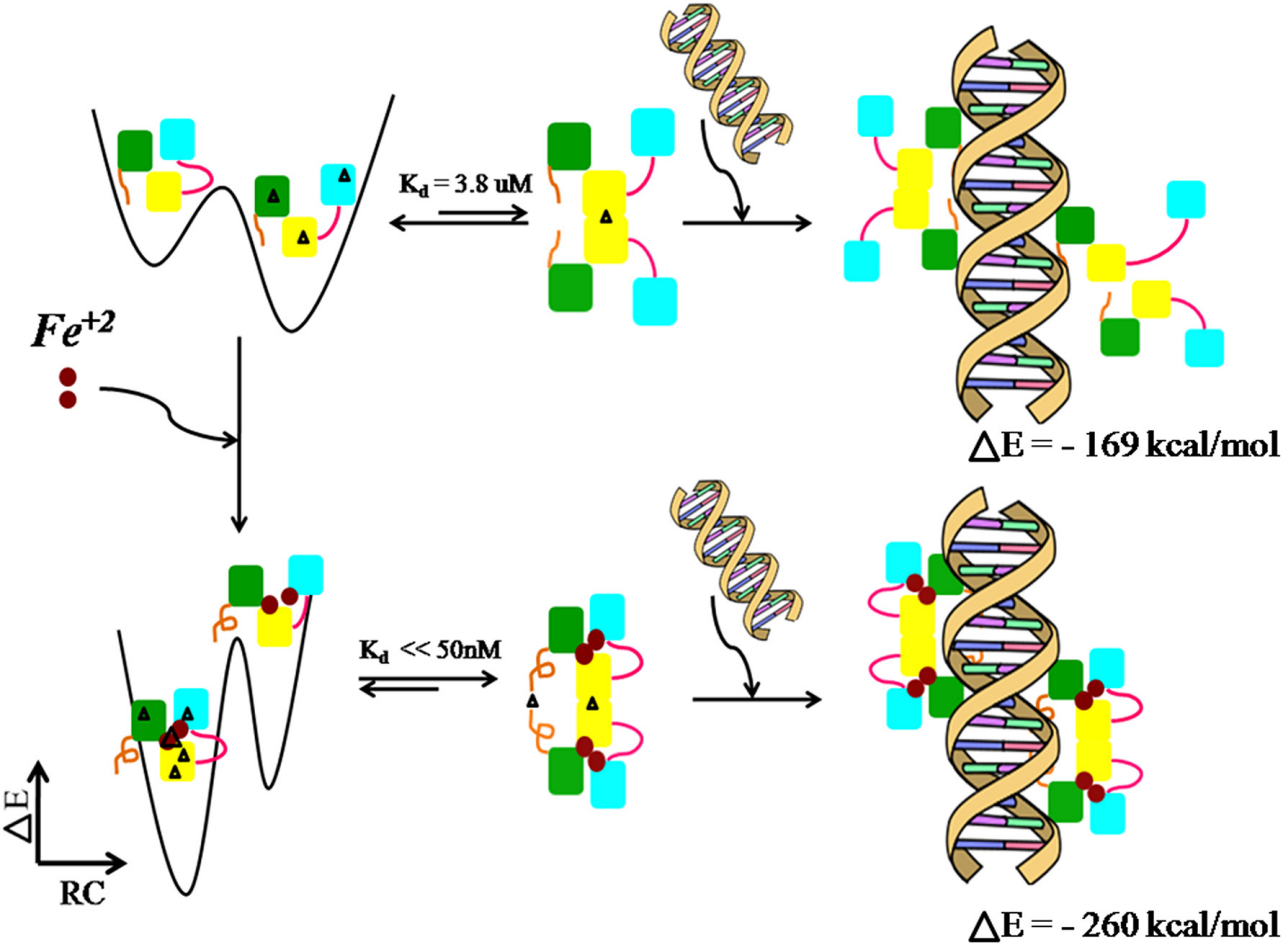
Proposed model of IdeR activation and DNA binding. A schematic representation of the various steps involved in IdeR activation as DNA binding as understood from experimental as well as simulations studies is provided. The three domains of IdeR monomer are represented in different colours, green–DNA binding domain, yellow–dimerization domain, and cyan–SH3 domain. The N-terminal region is shown in brown and the linker region is represented in magenta. The black triangles in the structure represent network connectivity as obtained from Protein Structure Network analysis. The top three panels correspond to the population distribution of the ‘open’ and ‘closed’ states of IdeR in the monomeric form, cartoon diagram of the dimeric form, and the DNA complexed with four units of IdeR, in the absence of metal ion. The three panels in the bottom row correspond to the same in the presence of metal ion.

The various experimental studies performed on IdeR as well as the simulation of the different phases of IdeR structure carried out in this study have enabled us to integrate the different aspects of structure dynamics and interaction to propose a model of IdeR activation and DNA binding (Fig 8). Parts of the model were understood from previous studies and have been refined here with more details. The model incorporates the following points. It was previously known from kinetic studies that IdeR exists as monomer-dimer equilibrium, with the equilibrium shifted more towards the monomer in the absence of metal. Simulation studies on IdeR monomer have now indicated that the monomer can take up two conformations, the ‘open’ and the ‘close’ conformation. In the absence of metal, continuous transition exists between the two conformations, while a shift in the conformational landscape is observed upon iron binding. Protein structure network analysis and interdomain angle analyses suggest that the metallated system prefers the ‘close’ conformation more than the ‘open’ conformation. Additionally, network connectivity of the metallated system indicates a rigid structure, mainly at the junction region and the dimerization domain. On the other hand, flexibility is observed in the DNA binding helices upon iron binding. Moving on to the dimers, it is noted that metal binding stabilizes the N-terminal residues and shifts the location of dimerization. It also influences the orientation of the DNA binding helix to favourably position the helix to interact with DNA. Finally, the IdeR-DNA complex is found to be more stable in the presence of iron as compared to the unmetallated complex. In FU-nm, two types of motions are observed, the rigid-body motion that results in movement of the complete monomeric subunit away from the DNA and the SH3 domain movement that results in the ‘open’ and ‘close’ conformations. The energetic difference between the two complexes is ~100kcal/mol as calculated using the MMPBSA analysis. All the above observations clearly highlight the critical role of iron for IdeR activation and function, with every step involved in IdeR activation and DNA binding being driven by iron. A striking point to note is that the structural modifications taken up by the protein at each step appear to be aimed towards performing its physiological function of DNA binding. Furthermore, the asymmetry in binding of IdeR to DNA, as presented from simulations, enhances our understanding of the ‘hemispecific’ nature of binding observed by experiments [11,13]. Thus, the model depicted in Fig 8 provides a framework for further investigations to understand the functioning of IdeR.

To conclude, an atomistic level study on IdeR, an iron responsive transcription factors have been performed to understand the mode of iron-dependent activation of IdeR and DNA binding, using a combination of molecular dynamics and protein structure network approaches. Our studies have successfully established the presence of two major monomeric conformations, the ‘open’ and ‘closed’ conformations that are preferred by the protein under different iron bound conditions. Additionally, the apo form of the monomer is also found be much more flexible than its metallated counterpart. We believe that the inherent flexibility observed probably provides the structure with an added advantage to search for metal ions in the vicinity and finally to accommodate ligand binding. Once both the metal ions are bound to the structure, the conformational flexibility of IdeR monomer decreases drastically. The role of N-terminal residues in DNA binding has also been discussed in great details and qualitatively as well as quantitatively validated.

The protein-DNA interface has also been characterized based on hydrogen bond formation and non-covalent interactions. Instability in the IdeR-DNA complex in the absence of iron can be easily attributed to these interactions. In the metallated system, protein residues from the DBH, 2^nd^ helix as well as the N-terminal residues were seen to contribute to complex formation. Although the individual monomeric subunits of IdeR do not behave identically, it is now understood that formation of a stable IdeR-DNA complex requires contribution from the DBH, 2^nd^ helix and the N-terminal residues. Most importantly, investigation of the dynamic properties of IdeR-DNA interactions has enabled us to study the mode of interaction between IdeR and DNA, with special focus to allostery. A brief discussion on the mechanism of dissociation or association of the IdeR-DNA complex has also been provided.

Finally, based on our simulations and previous studies we have proposed a model of IdeR activation and DNA binding. Models such as this could provide useful insights for drug designing and for general understanding of protein-DNA interactions.

## Methods

To obtain a detailed understanding of IdeR functioning and its interaction with DNA, different states of IdeR structure were analyzed. Details of these structures are provided in Table 1. The initial steps involve metal-site parameterization and molecular dynamics simulation. Further, the trajectories are analyzed for essential modes of motion[33] and population based free energy landscapes[34]. The dynamics at the residue level interactions were examined by hydrogen bond and protein structure networks[35]. The details of each of these steps are presented below.

### Metal site parameterization

Analysis of large sets of metalloproteins have identified the versatility of metal binding sites, with the metal ion binding to different sets of residues and forming different geometries of the coordinate complexes [36]. These geometries are unique to a given protein and hence *ab-intio* parameterization is required.

In this study, modified partial charges for the two metal centers of IdeR, and force constants were obtained by subjecting the metal centers to *ab-initio* calculations using Metal Center Parameter Builder (MCPB) [37] and Gaussian 09 [38]. The local environment or the metal center is first minimized followed by charge calculations to obtain the Merz-Singh-Kollman(MK) charges at the B3LYP/6-311G level of basis set. The partial charges were processed and finally integrated into AMBER force-field to perform the simulations and has been used throughout the study. The modified charge distribution for the two metal centers is listed in Table A of S3 Text. The coordinates of the metal centers used to generate the partial charges is also provided in Table G in S3 Text.

### Molecular dynamics simulations

PDB structures, 1FX7 [9] and 1U8R[10] have been used as the starting structures for the simulations of IdeR and IdeR-DNA complex, respectively. For the iron bound simulations, modified partial charges as calculated using MCPB have been used. Molecular Dynamics (MD) simulations were carried out using the GPU compatible PMEMD engine of AMBER 11 [39] and AMBER ff99SB force-fields [40]. Explicit water molecules were used. The solvation box was built such that a buffer area of 12Å was maintained between the protein and box boundary. The simulation protocol is summarized in Table B in S3 Text. Briefly, the crystal structures were minimized using 500 steps of ‘Steepest Descent’ followed by 2500 steps of ‘Conjugate Gradient’. The minimized structures are then equilibrated in three phases, a) solvent equilibration keeping the solute fixed for 20 ps, b) temperature ramping for 50 ps keeping the metal centers (MS1 and MS2) fixed and finally, c) 50 ps of equilibration. In totality, equilibration was performed for 120 ps. This was followed by unconstrained production run for 100 ns at 300K temperature in aqueous medium using the TIP3P water model at constant pressure ensemble and periodic boundary conditions. Particle mesh Ewald summation [41] was used for long-range electrostatics and the van der Waals cutoff was 10 Å. A time step of 2.0 fs was employed with the integration algorithm, and structures were stored every 10 ps. The Shake algorithm [42] was used to constrain covalently bound hydrogen atoms.

Given that the simulations were performed to assess the role of iron in IdeR, the geometry of the metal centers were monitored throughout the simulations. Correct geometry of the metal centers would imply that the effect seen on IdeR were indeed a result of iron binding and not a result of structural destabilization or modification. S8 Fig and S9 Fig provides the distribution plot of representative parameters that defines the geometry of the metal centers. The distribution plot represents the deviations in the values over the course of the simulations. Additionally, Table F in S3 Text provides the mean and standard deviation of all the parameters and compares them with known experimental values. As can be seen from the distribution plots as well as the table, the values of the different parameters (angle and distance between Fe and the coordinating ligands) observed in the simulations are mostly equivalent. A significant deviation of ~10° is observed in the angles of the coordination sphere of MS2, when compared to the ideal values. This has also been observed in the crystal structure of IdeR [9], where the values are seen to deviate slightly than the ideal 90°. Overall, the distribution plots and the table clearly indicate that the geometry of the metal centers (MS1 and MS2) has been maintained throughout the simulation in all the metallated system and that the effects observed in the protein structure are a result of the presence or absence of iron.

### Essential dynamics, conformational re-distribution and statistical free energy

Essential dynamics is a powerful tool to identify the ‘essential’ subspace of protein dynamics by reducing them into its essential degrees of freedom [33]. For each system, snapshots were obtained at every 10 ps of the last 51 ns simulations and aligned to the average MD structure to obtain the covariance matrix of positional fluctuation. Structures are aligned using the C^α^ atom and fluctuations calculated using the position coordinates of the atom (Eq 1).

$$C_{ij}=\frac{1}{S}\sum_{t}\left[(x_{i}(t)-\left\langle x_{i}\right\rangle )(x_{j}(t)-\left\langle x_{j}\right\rangle )\right]$$ (1)

where, S is the total number of snapshots, t is 1, 2,3… S, ⟨x_i_⟩ represents the average position over all snapshots, x_i_(t) is the position coordinate of snapshot ‘t’ with i ranging from 1 to 3N (where, N is the number of atoms (C^α^ or all atoms using which C is constructed)).

The covariance matrix is then diagonalized to obtain a set of eigenvalues and eigenvectors. While the eigenvectors define the nature of movements, the corresponding eigenvalues define the amount of variation in each movement. Hence, sorting the eigenvalues in the descending order provides a listing of the major motions in the structure. Additionally, the relative cumulative positional fluctuation (RCPF) can also be measured [33]using Eq 2. This provides an estimate of the minimum number of eigenvectors required to describe the major conformational motion in the structures.

$$RCPF(n)=\frac{\sum_{i=1,n}\lambda_{i}}{\sum_{i=1,3n}\lambda_{i}}$$ (2)

In our study, we have considered two principal components with the highest eigenvalues to define the “essential plane” for our analysis. A quantitative measure of the complexity of a system can be obtained from normalized eigenvalues distribution[43]. However, a simplistic analysis of the top PCA values obtained from essential dynamics can capture major part of the global dynamics of the system[33]. Studies have shown that analysis of the simulation trajectories can divide the protein motions into two subspaces. One of these define the “essential” subspace and comprise of positional fluctuations important for the function of the protein. These are generally defined by the top two/three eigen values. The remaining eigen vectors represent the local fluctuations that are a result of fluctuations that are “physically constrained” due to covalent interactions between atoms. Such a division of subspace enables a reduction in the subspace and allows focusing on motions that are important for protein function [34,44].

The “essential plane” is further divided into a grid of size n x n, and conformations sampled at every 10ps are binned into the plane to obtain a population distribution profile using MATLAB. For each cell, relative probabilities are calculated with reference to the cell containing maximum number of projections for calculation of Helmholtz free energy utilized in moving from the i^th^ cell to the reference cell (Eq 3).

$$\Delta A_{i\to ref}=-RT\ln\frac{p_{i}}{p_{ref}}$$ (3)

where, *R* is ideal gas constant, *T* is temperature (300 K), and p_i_ and p_ref_ are the probabilities of finding the system in the *i* ^th^ cell and the reference cell, respectively.

Eigenvectors corresponding to the top eigenvalue are also projected on the structure to generate a porcupine plot, using PCAzip[44].

### Dynamically stable hydrogen bonds

Hydrogen bonds are known to be crucial for the secondary and tertiary structure of any protein. Given the conformational flexibility of a protein, some of the hydrogen bonds are not necessarily stable and may change over the course of simulations. Thus, hydrogen bond dynamics are studied here for 100ns simulations, using a distance cut-off of 3.5Å between the donor and the acceptor atoms and an angle cut-off of 120°.

### Protein Structure Networks

In this study, protein structure networks (PSNs) are generated at the level of non-covalent sidechain interactions using the technique defined in[35]. The networks are constructed by considering residues as nodes and edges are formed between them if a sidechain atom pair exists within a cutoff distance of 4.5Å. For each pair of residues, interaction strengths (I_ij_) are calculated as shown below to account for the strength of non-covalent interaction between any two residues.

$$I_{ij}=\frac{n_{ij}}{\sqrt{N_{i}\times N_{j}}}\times 100$$ (4)

where, I_ij_ = Interaction energy between residue i and j; n_ij_ = number of atom pairs within the cutoff distance; N_i/j_ = Normalization value of residue i and j. In this study, PSNs are generated for snapshots at every 10ps at I_min_ = 4%, unless mentioned otherwise.

For protein-DNA interactions, a variant of protein sidechain network, protein-DNA graphs (PDG) that focuses on the interaction between protein and DNA have been used [45]. In this representation, a bipartite graph is constructed with protein and DNA residues representing two sets of nodes. Edges are defined only for the connection between a DNA and a protein residue and also when the strength of the interaction is greater than a user defined cutoff, termed as the Minimal Effective Connection (MEC). MEC quantifies the minimum number of atomic contacts expected between an amino acid and a nucleotide and can range from 0% to 15%, representing weak to strong interactions. It is observed that the different components of DNA (bases, phosphate group and deoxyribose) show different strengths of interactions and hence the interaction strength of these components with protein residues should be evaluated independently [45]. In this study, PDGs are generated to represent interactions with the three DNA components at MEC = 2%.

### Dynamically stable network parameters

The PSNs constructed are analyzed in terms of dynamically stable parameters. Clusters are identified using the depth first search algorithm [46] and further evaluated to identify interface clusters, formed by residues belonging to multiple chains. The communities in the structures represent regions of higher connectivity in a PSN and have been evaluated in this study for identification of rigid regions of the protein. The program CFinder [47] is employed for detection of communities. All the network parameters are considered significant if they occur in at least 70% of the snapshots and hence are dynamically stable. The above analysis is performed in an automated manner using PSN-Ensemble [48], a software developed in the laboratory.

### Binding energy calculations using ensemble structures

The simulation trajectories are also used to calculate the free energy of complex formation, using the MMPBSA module of AMBER 11[39]. MMPBSA is a post-processing method that uses the ensemble of complex structures from MD simulation to derive the free energy of binding. It is based on the key assumption that the free energy of binding result from the additive contribution of individual components as explained in Eq 5.

$$\Delta G_{bind}=\Delta G_{AB}-(\Delta G_{A}+\Delta G_{B})$$ (5)

MMPBSA uses an implicit solvent model to calculate the binding energy. Additionally, contribution of each residue pair towards the free energy of complex formation has also been calculated in this study.

## Supporting Information

S1 Text IdeR monomers exhibit conformational flexibility: The ‘open’ and ‘close’ conformations. (PDF)

S2 Text Iron binding stabilizes N-terminal residues in IdeR dimer. (PDF)

S3 Text Supplementary tables. (PDF)

S1 Fig Structural details of the metal binding sites are shown for a) metal site 1 [MS1], b) metal site 2 [MS2]. MS1 forms a distorted bipyramidal geometry using sidechain atoms of five residues, while MS2 forms an octahedral geometry using sidechain atoms of four residues and a water molecule. In this case, Cys102 provides two ligands to the metal ion. Each residue is colour coded based on the domain, to which they belong, [green: DBD, yellow: DD, pink: linker and cyan: SH3 like domain].
(TIF)

S2 Fig RMSD profile and residue level fluctuations for the three monomeric systems. a) Cα RMSD profile of the three monomeric systems, M, M1 and M2 for 100 ns simulations b) Residue wise fluctuation for each system. Residue numbers are also mapped to individual domains.
(TIF)

S3 Fig Porcupine plots showing the essential modes of the three monomeric systems. Eigen vectors corresponding to the first eigen value are projected on a) no iron bound form [M], b) Fe bound at MS1 [M1] and c) iron bound at both MS1 and MS2 [M2]. The red arrows indicate the direction as well as the magnitude of motions in the systems. Based on the domain movements, the structures are termed as the ‘open’ and the ‘closed’ conformations.
(TIF)

S4 Fig RMSD profile for the DNA bound complexes. a) Cαbackbone RMSD of the protein subunits of the FU-nm/m cases are plotted over the 100 ns simulations The large variation observed for FU-nm is discussed in details in the text. b) All atom RMSD of the DNA subunit is plotted as a function of time.
(TIF)

S5 Fig Orientation of Gln43 in the different monomeric subunits of FU-m. Gln43 is the only residue that interacts with a nucleobase, however not in all subunits. This interaction is guided by the location of Gln43 and base accessibility. Figure represents the side chain orientation of Gln43 in all the subunits and is shown in a stick representation. The DNA binding Helix is shown as green cylinders, while DNA is represented in orange.
(TIF)

S6 Fig a) Statistics of the hydrogen bond networks generated for FU-nm and FU-m. b) Routes taken up the 14 shortest paths in the hydrogen bond network of FU-m (last row of S6(a)) is mapped on the protein structure. Cascades of hydrogen bonds formed in chain A and B are highlighted.
(TIF)

S7 Fig DNA-DBH distance. a) DNA binding helices of the different chains and the interacting DNA residues are marked on the structure for representation. The centers of mass of the highlighted residues were used for calculating the DNA-DBH distance in b) FU-nm and c) FU-m.
(TIF)

S8 Fig Distribution plot representing the distance between Fe and its coordinating ligands for all the metallated systems. Top panel shows distance values for MS1 and the lower shows values for MS2. X-axis represents the distance values and the y-axis represents the percentage of snapshots with the given distance values. The values match well with experimental results as indicated in Table G of S3 Text.
(TIF)

S9 Fig Distribution plot representing the angle between Fe and its coordinating ligands for all the metallated systems. Top panel shows distance values for MS1 and the lower shows values for MS2. X-axis represent the angle values and the y-axis represent the percentage of snapshots with the given values.
(TIF)

## References

[1] World Health Organization W (2014) Global tuberculosis report 2014.

[2] Baynes RD, Flax H, Bothwell TH, Bezwoda WR, MacPhail AP, et al (1986) Haematological and iron-related measurements in active pulmonary tuberculosis. Scand J Haematol 36: 280–287. 3704552 10.1111/j.1600-0609.1986.tb01735.x

[3] Murray MJ, Murray AB, Murray MB, Murray CJ (1978) The adverse effect of iron repletion on the course of certain infections. Br Med J 2: 1113–1115. 361162 10.1136/bmj.2.6145.1113 PMC1608230

[4] Ratledge C (2004) Iron, mycobacteria and tuberculosis. Tuberculosis 84: 110–130. 14670352 10.1016/j.tube.2003.08.012

[5] Atamna H, Walter PB, Ames BN (2002) The role of heme and iron-sulfur clusters in mitochondrial biogenesis, maintenance, and decay with age. Arch Biochem Biophys 397: 345–353. 11795893 10.1006/abbi.2001.2671

[6] Ghosh S, Prasad KV, Vishveshwara S, Chandra N (2011) Rule-based modelling of iron homeostasis in tuberculosis. Molecular BioSystems 7: 2750–2768. 10.1039/c1mb05093a 21833436

[7] Barry CE, Boshoff H (2005) Getting the iron out. Nat Chem Biol 1: 127–128. 16408014 10.1038/nchembio0805-127

[8] Rodriguez GM, Voskuil MI, Gold B, Schoolnik GK, Smith I (2002) ideR, an essential gene in Mycobacterium tuberculosis: role of IdeR in iron-dependent gene expression, iron metabolism, and oxidative stress response. Infection and immunity 70: 3371–3381. 12065475 10.1128/IAI.70.7.3371-3381.2002 PMC128082

[9] Feese MD, Ingason BP, Goranson-Siekierke J, Holmes RK, Hol WG (2001) Crystal structure of the iron-dependent regulator from Mycobacterium tuberculosis at 2.0-A resolution reveals the Src homology domain 3-like fold and metal binding function of the third domain. J Biol Chem 276: 5959–5966. 11053439 10.1074/jbc.M007531200

[10] Wisedchaisri G, Holmes RK, Hol WG (2004) Crystal structure of an IdeR–DNA complex reveals a conformational change in activated IdeR for base-specific interactions. Journal of molecular biology 342: 1155–1169. 15351642 10.1016/j.jmb.2004.07.083

[11] Chou CJ, Wisedchaisri G, Monfeli RR, Oram DM, Holmes RK, et al (2004) Functional studies of the Mycobacterium tuberculosis iron-dependent regulator. Journal of Biological Chemistry 279: 53554–53561. 15456786 10.1074/jbc.M407385200

[12] Ding X, Zeng H, Schiering N, Ringe D, Murphy J (1996) Identification of the primary metal ion-activation sites of the diphtheria tox represser by X-ray crystallography and site-directed mutational analysis. Nature Structural & Molecular Biology 3: 382–387. 10.1038/nsb0496-382 8599765

[13] Townson SA, Samuelson JC, Bao Y, Xu S-y, Aggarwal AK (2007) BstYI bound to noncognate DNA reveals a “hemispecific” complex: implications for DNA scanning. Structure 15: 449–459. 17437717 10.1016/j.str.2007.03.002

[14] Floyd RW (1962) Algorithm 97: shortest path. Communications of the ACM 5: 345.

[15] Cui Q, Karplus M (2008) Allostery and cooperativity revisited. Protein science 17: 1295–1307. 10.1110/ps.03259908 18560010 PMC2492820

[16] Monod J, Wyman J, Changeux J-P (1965) On the nature of allosteric transitions: a plausible model. Journal of molecular biology 12: 88–118. 14343300 10.1016/s0022-2836(65)80285-6

[17] Koshland D Jr, Nemethy G, Filmer D (1966) Comparison of Experimental Binding Data and Theoretical Models in Proteins Containing Subunits*. Biochemistry 5: 365–385. 5938952 10.1021/bi00865a047

[18] Perutz M (1970) Stereochemistry of cooperative effects in haemoglobin: haem–haem interaction and the problem of allostery. 10.1038/228726a0 5528785

[19] Perutz MF, Wilkinson A, Paoli M, Dodson G (1998) The stereochemical mechanism of the cooperative effects in hemoglobin revisited. Annual review of biophysics and biomolecular structure 27: 1–34. 9646860 10.1146/annurev.biophys.27.1.1

[20] Frauenfelder H, McMahon BH, Austin RH, Chu K, Groves JT (2001) The role of structure, energy landscape, dynamics, and allostery in the enzymatic function of myoglobin. Proceedings of the National Academy of Sciences 98: 2370–2374. 10.1073/pnas.041614298 PMC30145 11226246

[21] Motlagh HN, Wrabl JO, Li J, Hilser VJ (2014) The ensemble nature of allostery. Nature 508: 331–339. 10.1038/nature13001 24740064 PMC4224315

[22] Gunasekaran K, Ma B, Nussinov R (2004) Is allostery an intrinsic property of all dynamic proteins? Proteins: Structure, Function, and Bioinformatics 57: 433–443. 10.1002/prot.20232 15382234

[23] Popovych N, Sun S, Ebright RH, Kalodimos CG (2006) Dynamically driven protein allostery. Nature structural & molecular biology 13: 831–838. 16906160 10.1038/nsmb1132 PMC2757644

[24] Hammes GG, Benkovic SJ, Hammes-Schiffer S (2011) Flexibility, diversity, and cooperativity: pillars of enzyme catalysis. Biochemistry 50: 10422–10430. 10.1021/bi201486f 22029278 PMC3226911

[25] Szilagyi A, Nussinov R, Csermely P (2013) Allo-network drugs: extension of the allosteric drug concept to protein- protein interaction and signaling networks. Curr Top Med Chem 13: 64–77. 23409766 10.2174/1568026611313010007

[26] Kumar P, Kailasam S, Chakraborty S, Bansal M (2014) MolBridge: a program for identifying nonbonded interactions in small molecules and biomolecular structures. Applied Crystallography 47.

[27] Reichheld SE, Yu Z, Davidson AR (2009) The induction of folding cooperativity by ligand binding drives the allosteric response of tetracycline repressor. Proceedings of the National Academy of Sciences 106: 22263–22268. 10.1073/pnas.0911566106 PMC2799725 20080791

[28] Schrank TP, Bolen DW, Hilser VJ (2009) Rational modulation of conformational fluctuations in adenylate kinase reveals a local unfolding mechanism for allostery and functional adaptation in proteins. Proceedings of the National Academy of Sciences 106: 16984–16989. 10.1073/pnas.0906510106 PMC2761315 19805185

[29] Bhattacharyya M, Ghosh A, Hansia P, Vishveshwara S (2010) Allostery and conformational free energy changes in human tryptophanyl‐tRNA synthetase from essential dynamics and structure networks. Proteins: Structure, Function, and Bioinformatics 78: 506–517. 10.1002/prot.22573 19768679

[30] Ghosh A, Vishveshwara S (2008) Variations in Clique and Community Patterns in Protein Structures during Allosteric Communication: Investigation of Dynamically Equilibrated Structures of Methionyl tRNA Synthetase Complexes†. Biochemistry 47: 11398–11407. 10.1021/bi8007559 18842003

[31] Bhattacharyya M, Vishveshwara S (2011) Probing the allosteric mechanism in pyrrolysyl-tRNA synthetase using energy-weighted network formalism. Biochemistry 50: 6225–6236. 10.1021/bi200306u 21650159

[32] Goodey NM, Benkovic SJ (2008) Allosteric regulation and catalysis emerge via a common route. Nat Chem Biol 4: 474–482. 10.1038/nchembio.98 18641628

[33] Amadei A, Linssen AB, Berendsen HJ (1993) Essential dynamics of proteins. Proteins 17: 412–425. 8108382 10.1002/prot.340170408

[34] Daidone I, Amadei A, Di Nola A (2005) Thermodynamic and kinetic characterization of a beta-hairpin peptide in solution: an extended phase space sampling by molecular dynamics simulations in explicit water. Proteins 59: 510–518. 15789436 10.1002/prot.20427

[35] Kannan N, Vishveshwara S (1999) Identification of side-chain clusters in protein structures by a graph spectral method. Journal of molecular biology 292: 441–464. 10493887 10.1006/jmbi.1999.3058

[36] Babor M, Greenblatt HM, Edelman M, Sobolev V (2005) Flexibility of metal binding sites in proteins on a database scale. Proteins: Structure, Function, and Bioinformatics 59: 221–230. 10.1002/prot.20431 15726624

[37] Peters MB, Yang Y, Wang B, Füsti-Molnár Ls, Weaver MN, et al (2010) Structural survey of zinc-containing proteins and development of the zinc AMBER force field (ZAFF). Journal of chemical theory and computation 6: 2935–2947. 20856692 10.1021/ct1002626 PMC2941202

[38] Frisch GWT M. J., Schlegel H. B., Scuseria G. E., Robb M. A., Cheeseman J. R., Scalmani G., Barone V., Mennucci B., Petersson G. A., Nakatsuji H., Caricato M., Li X., Hratchian H. P., Izmaylov A. F., Bloino J., Zheng G., Sonnenberg J. L., Hada M., Ehara M., Toyota K., Fukuda R., Hasegawa J., Ishida M., Nakajima T., Honda Y., Kitao O., Nakai H., Vreven T., Montgomery J. A. Jr., Peralta J. E., Ogliaro F., Bearpark M., Heyd J. J., Brothers E., Kudin K. N., Staroverov V. N., Kobayashi R., Normand J., Raghavachari K., Rendell A., Burant J. C., Iyengar S. S., Tomasi J., Cossi M., Rega N., Millam J. M., Klene M., Knox J. E., Cross J. B., Bakken V., Adamo C., Jaramillo J., Gomperts R., Stratmann R. E., Yazyev O., Austin A. J., Cammi R., Pomelli C., Ochterski J. W., Martin R. L., Morokuma K., Zakrzewski V. G., Voth G. A., Salvador P., Dannenberg J. J., Dapprich S., Daniels A. D., Farkas Ö., Foresman J. B., Ortiz J. V., Cioslowski J., and Fox D. J., Gaussian, Inc., Wallingford CT (2009) Gaussian 09, Revision D.01.

[39] Case De, Darden T, Cheatham T, Simmerling C, Wang J, et al (2010) Amber 11. University of California.

[40] Hornak V, Abel R, Okur A, Strockbine B, Roitberg A, et al (2006) Comparison of multiple Amber force fields and development of improved protein backbone parameters. Proteins: Structure, Function, and Bioinformatics 65: 712–725. 10.1002/prot.21123 PMC4805110 16981200

[41] Darden T, York D, Pedersen L (1993) Particle mesh Ewald: An N⋅ log (N) method for Ewald sums in large systems. The Journal of chemical physics 98: 10089–10092.

[42] Ryckaert J-P, Ciccotti G, Berendsen HJ (1977) Numerical integration of the cartesian equations of motion of a system with constraints: molecular dynamics of n-alkanes. Journal of Computational Physics 23: 327–341.

[43] Giuliani A, Colafranceschi M, Webber CL, Zbilut JP (2001) A complexity score derived from principal components analysis of nonlinear order measures. Physica A: Statistical Mechanics and its Applications 301: 567–588.

[44] Meyer T, Ferrer-Costa C, Pérez A, Rueda M, Bidon-Chanal A, et al (2006) Essential dynamics: a tool for efficient trajectory compression and management. Journal of Chemical Theory and Computation 2: 251–258. 26626512 10.1021/ct050285b

[45] Sathyapriya R, Vijayabaskar M, Vishveshwara S (2008) Insights into Protein–DNA Interactions through structure network analysis. PLoS computational biology 4: e1000170 10.1371/journal.pcbi.1000170 18773096 PMC2518215

[46] Cormen TH, Leiserson CE, Rivest RL, Stein C (2001) Introduction to algorithms: MIT press Cambridge.

[47] Adamcsek B, Palla G, Farkas IJ, Derényi I, Vicsek T (2006) CFinder: locating cliques and overlapping modules in biological networks. Bioinformatics 22: 1021–1023. 16473872 10.1093/bioinformatics/btl039

[48] Bhattacharyya M, Bhat CR, Vishveshwara S (2013) An automated approach to network features of protein structure ensembles. Protein Science 22: 1399–1416. 10.1002/pro.2333 23934896 PMC3795498

