# Supplementary material for: Mechanism of Iron-Dependent Repressor (IdeR) Activation and DNA Binding: A Molecular Dynamics and Protein Structure Network Study
Source: PLoS Comput Biol. 2015 Dec 23;11(12):e1004500. doi: 10.1371/journal.pcbi.1004500 (PMC4689551; doi:10.1371/journal.pcbi.1004500)
Supplement: S1 Text — (PDF) [file pcbi.1004500.s001.pdf]

S1_Text: IdeR monomers exhibit conformational flexibility: the 'open' and 'close' conformations

A quantitative characterization of the two conformations is performed by calculating the angle between the centers of mass of the three domains. Figure A shows a schematic representation of the interdomain angle calculated and its values over the entire simulations for the three monomeric systems. As compared to the two metallated form, the apo form shows larger fluctuations in the interdomain angle, indicating continuous fluctuations between the 'open' and 'close' conformations. On the other hand, the metallated system restricts themselves to a narrow range of interdomain angle, which in our simulation corresponds to the 'close' conformation. Such transitions have also been observed in the IdeR homologous protein, DtxR (26), where authors have suggested the interaction of SH3 domain with a proline-rick pocket [R125 – G139] in the dimerization domain to be important for the formation of the 'close' conformation. The proline-rich pocket [NPIPG] is conserved in the DtxR family of protein; however, interaction of the SH3 domains with this segment is not observed in our simulation studies. Instead, we recognize two factors that may be responsible for the shift in conformation upon iron binding, a) dynamics of the proline-rich [GPEPG] linker region [141-150] and b) involvement of residues Glu172 and Gln175 from SH3 domain along with three residues [His79, Glu83 and His98] from DBD for metal binding at MS1. Figure B illustrates the differences observed in the linker region and the hydrogen bond pattern between the two conformations.

To understand the differences in the linker region between the two conformations, we picked up two specific snapshots that exhibited extreme interdomain angle values (~85 and ~65°) and best represented the two conformations. Comparison of the linker region between the two structures, suggests an increased folding of the C-terminal end of the linker [Asp147, Asp148, Ala149 and Asn150] region in the 'open' conformation [Figure B (a)]. Linker corresponding to the 'closed' conformation does not undergo any folding, also indicated by the larger end-to-end length of the linker region [23.1 Å] for the 'closed' conformation as compared to the 'open' conformation [20.6 Å]. We believe that the relative organization of the three domains restrains the structure so that a longer linker is required for the SH3 domain to lie between the DBD and the DD, forming the 'closed' conformation, while shortening of the linker results in the 'open' conformation. Folding/unfolding of the linker region helps in controlling the length of the linker and hence the different conformations that the structure can take up. Secondary structure calculations of the linker region indicate higher degree of folding [increased formation of 1-hydrogen bonded turn] in the apo structure as compared to the iron bound structure. In addition to linker length, residues Glu172 and Gln175 also interact with the metal ion in the iron bound structure and help in bringing the SH3 domain closer to the DD. Infact, specific hydrogen bonds between residues Glu172 and Asn130 and residues Glu20 and Asn130 are observed only in the metallated system. These hydrogen bonds are strategically located at the junction region and bring the three domains closer [Figure B (b)].

a)

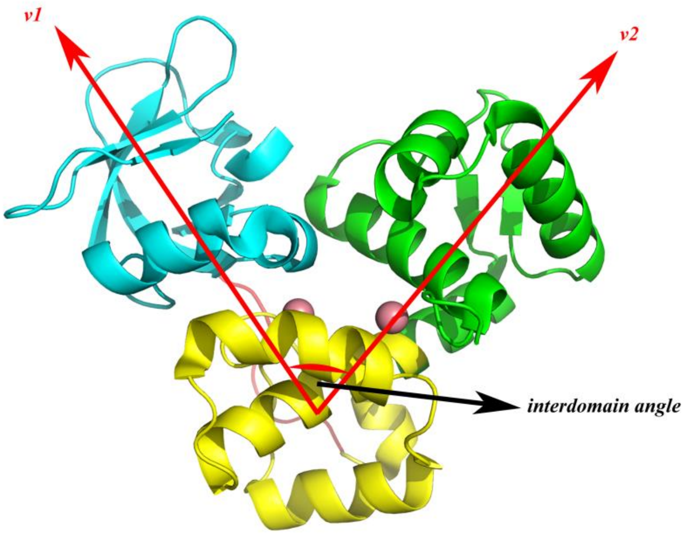

b)

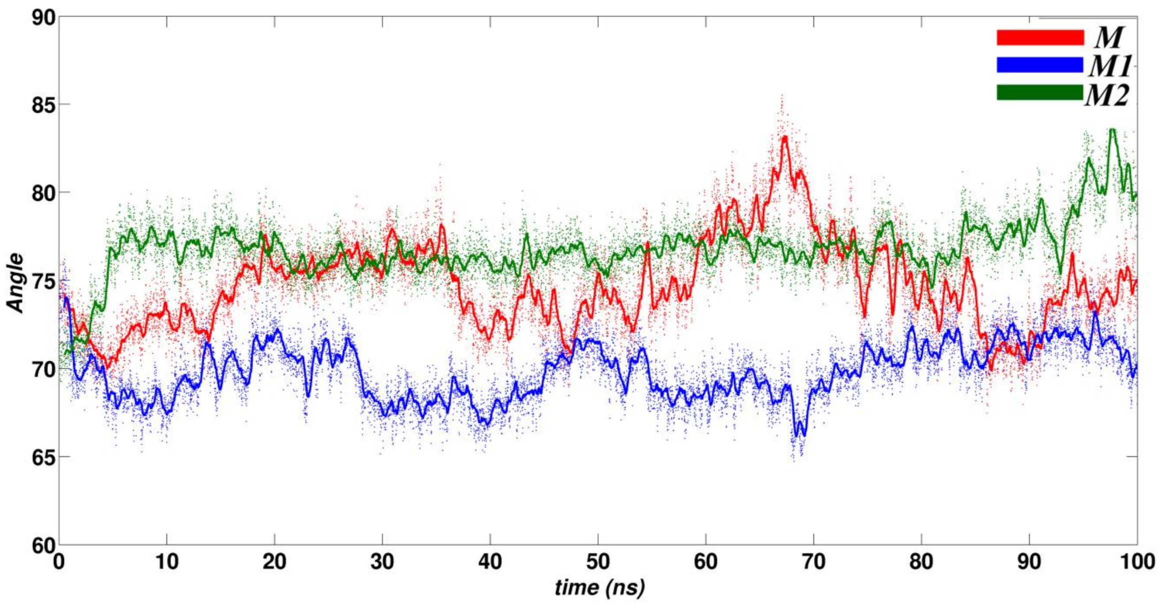

Figure A: Interdomain angle variations in the three monomeric systems are plotted. a) Schematic representation of the angle used to measure the interdomain movements b) Variation in the interdomain angle for the three monomeric systems over the entire simulation.

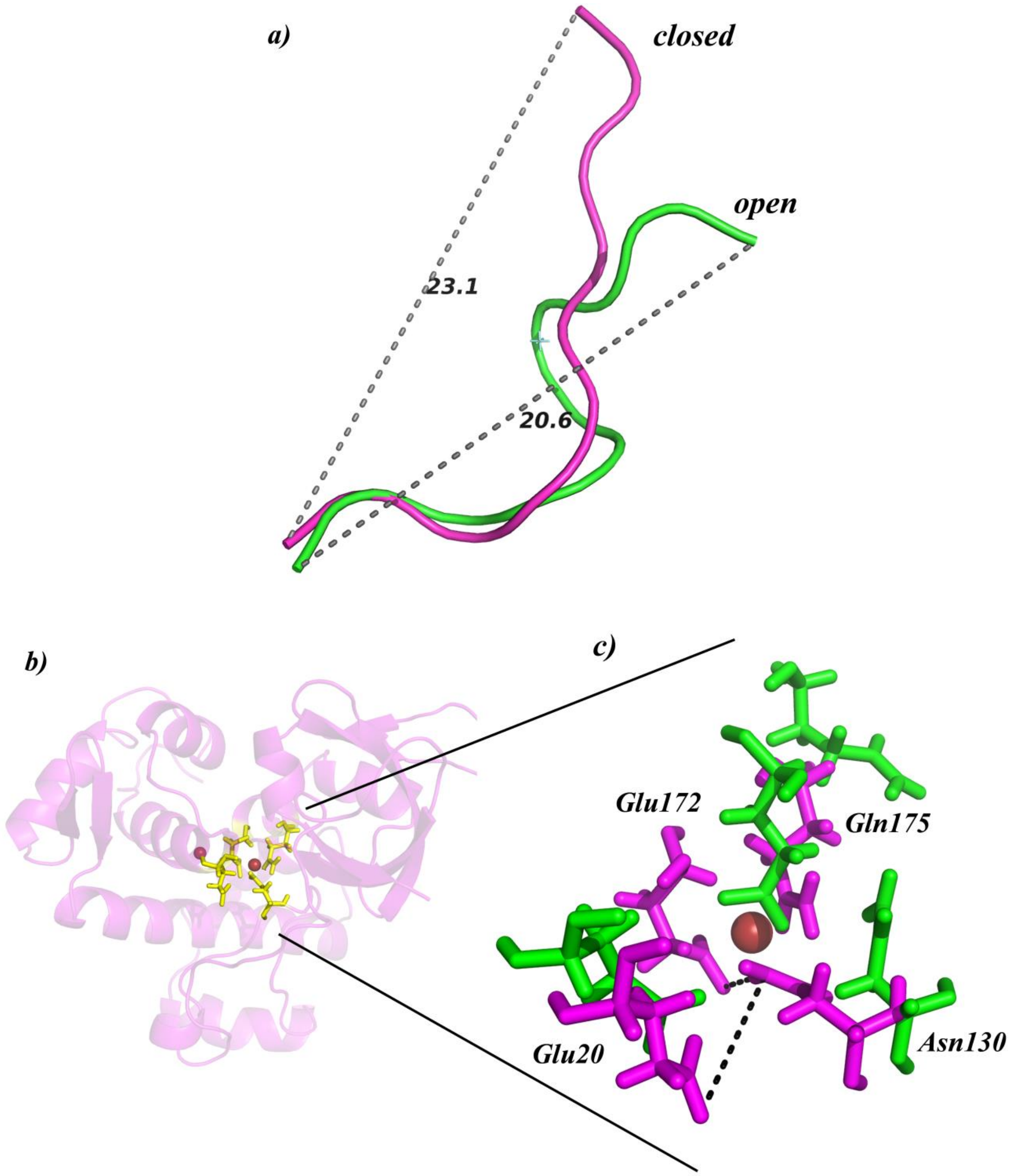

Figure B: Difference between the 'open' and 'close' conformations is demonstrated by a) superposition of the linker region from the 'open' [green] and 'close' [magenta] conformations, b) Formation of specific hydrogen bonds only in the 'close' conformation at the junction region and c) Enlarged version to clearly demonstrate hydrogen bonds formation between Glu172 – Asn130 and Glu20 – Asn130 in the 'close' conformation [magenta]. Further, Glu172 and Gln175 are also pulled towards the junction region [shown in magenta].
