# Supplementary material for: Mechanism of Iron-Dependent Repressor (IdeR) Activation and DNA Binding: A Molecular Dynamics and Protein Structure Network Study
Source: PLoS Comput Biol. 2015 Dec 23;11(12):e1004500. doi: 10.1371/journal.pcbi.1004500 (PMC4689551; doi:10.1371/journal.pcbi.1004500)
Supplement: S2 Text — (PDF) [file pcbi.1004500.s002.pdf]

S2_Text: Iron binding stabilizes N-terminal residues in IdeR dimer

To understand the influence of metal ions on IdeR dimers, we calculated dynamically stable interface clusters as obtained from protein structure networks. Figure A(a,b)shows the interface cluster distribution from the two systems at I_{min}= 4% and dynamic stability = 70%. Interestingly, a distinct break in symmetry is observed in the dimerization pattern of the metallated system. While the interface cluster in the non- metallated system is highly symmetric, formed by 3 identical residues (LEU86, TRP104 and Val107) from each chain, interface clusters in the metallated system show asymmetry with higher contribution from one of the monomeric subunits. Apart from the shift in the dimerization pattern, the interface cluster of the metallated system also suggests active involvement of N-terminal residues.These properties were further analysed at the residue level by calculating interface clusters for every snapshots in the simulation and calculating the propensity of residues to form interface clusters. Residue propensities were calculated at I_{min} = 4% and 6% (Figure A(c,f). Bar plots clearly indicate change in the dimerization pattern between the two cases. At I_{min} = 4%, active participation of residues from the dimerization domain is observed in the non-metallated system, while in the presence of metal ions, active participation of the N-terminal residues along with residues from the dimerization domainare observed. The residues with percentage occupancy > 40% were also mapped on the protein structure to visualize its location and understand the influence of iron. A shift in the location of dimerization upon iron binding is quite evident (Figure A(d,e)). At a still stronger interaction strength (I_{min} = 6%) the overall number of residues forming interface cluster reduces as compared to I_{min} = 4%. However, asymmetry between the two chains and persistence of N-terminal residue are clearly seen in the metal bound cases (Figure A(g,h)). The strength of dimerization and the energetic contribution of different residue pairs were also quantitatively estimated using MPBSA analysis of the last 51 ns of simulations. Residue pairs showing significant difference ($\Delta E > |5|$) between the metallated and the non-metallated systems are listed in Table A and their location on the protein structure is illustrated in Figure B. In correlation to the previous observations, the N-terminal residue pairs are seen to interact strongly in the presence of metal, while residue pairs from the dimerization domains contribute similarly towards dimer formation in both the systems. In summary, a shift in the location of dimerization is observed in the metallated system, with residues from the dimerization domain as well as the N-terminal region contributing towards dimer formation. This is in contrast to the non-metallated system, where residues from the dimerization domain participate in dimerization.

The influence of iron on the stability of N-terminal residues could be attributed to a cascade of hydrogen bonds that connects the metal binding residues [MBRs] to the N-terminal residues [1 to 9]. Table B provides a list of these consecutive hydrogen bond connections for both the dimers and Figure C illustrates the position of the hydrogen bonds on the protein structure. Between the metallated and the non-metallated cases, hydrogen bond profile of Met10 stands out. Met10 forms hydrogen bonds with the N-terminal residues in both the chains in the presence of iron and helps in holding the N-terminal residue in a particular position. Additionally, Met10 also forms coordinate bonds with the metal ion thereby restricting its position. Together, they reduce the flexibility of the N-terminal residues in the metallated system.

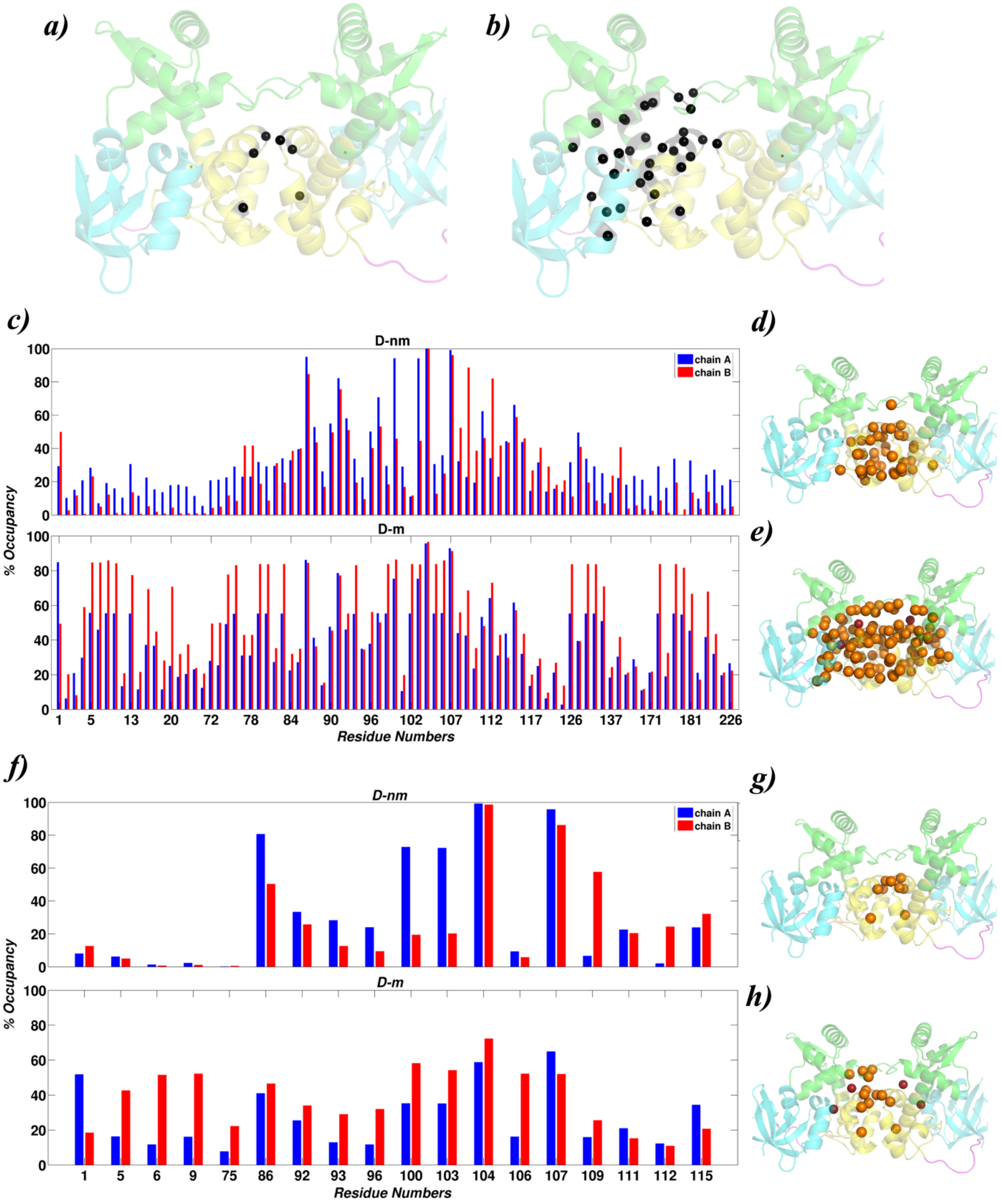
a)
b)
c)
D-nm
chain A
chain B
% Occupancy
D-m
1 5 13 20 72 78 84 90 96 102 107 112 117 126 137 171 181 226
Residue Numbers
d)
e)
f)
D-nm
chain A
chain B
% Occupancy
D-m
1 5 6 9 75 86 92 93 96 100 103 104 106 107 109 111 112 115
Residue Numbers
g)
h)

Figure A Figure illustrating the change in the dimerization pattern upon iron binding. Dynamically stable interface cluster, calculated at I_{min} = 4% and 70% dynamic stability is plotted for a) D-nm and b) D-m. Bar plots represents the percentage occupancy of a residue to be a part of the interface cluster at c) I_{min} = 4% and f) I_{min} = 6%. The two colours represent each monomeric subunit of IdeR dimer. X axis represents the residues, while Y axis represents the %occupancy of that particular residue in the interface cluster. Residues that show percentage occupancy > 40% are also mapped on the protein structures for d) D-nm at I_{min} = 4%, e) D-m at I_{min} = 4%, g) D-nm at I_{min} = 6% and h) D-m at I_{min}= 6%. Mapping of residues clearly indicate a shift in the location of dimerization.

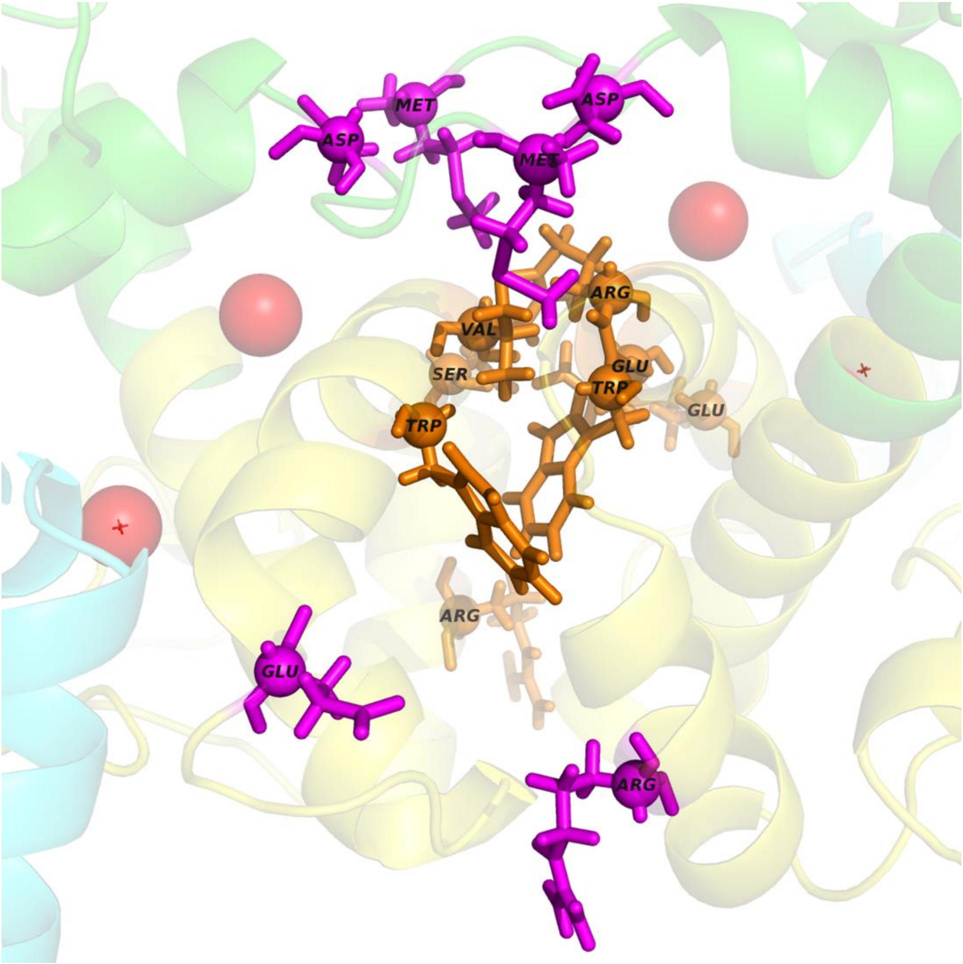

Figure B Pymol representation of the pair of residues showing different free energy contribution towards dimer formation, in the metallated and the non-metallated cases. Residues shown in magenta contribute significantly in the metallated case, and pairs shown in orange contribute significantly towards dimer formation in the non-metallated system. Analysis was performed using MMPBSA. List of pairs are provided in Table A. N-terminal residues contribute significantly in the metallated system while residues from the dimerization domain contribute favourably in the non metallated system.

Table A Residue pairs that have significantly different energetic contribution as calculated using MMPBSA between D-nm and D-m

Chain A	***Chain B***	***D-nm energy (kcal/mol)***	***D-m energy(kcal/mol)***
M 1	D 6	-0.0764	-7.8925
D 6	M 1	-0.2048	-1.3855
R 115	E 96	-0.3477	-10.6455
E 100	S 109	-4.9524	-0.9658
E 96	R 115	-2.4619	-0.3328
W 104	W 104	-2.7846	-0.7756
R 103	S 109	-0.6287	-1.9920
W 104	V 107	-3.6965	-2.3853

Table B Connections found in the shortest paths between metal binding residues and the N-terminal residues in the two dimeric systems are listed in the table.

Dimer –nm	***Dimer-m***
Asn2A→Leu4A→His106A	Asn2A→Leu4A→Asp6A→Met10A
Glu3A→Arg103A→His106A	Thr7A→Met10A
Glu3B→His106B	Asn2B→Leu4B→Asp6B→Met10B
Asp6B→Glu9B→Arg13B→ Asp17B→His173B→Thr171B→Gln175B	Glu3B→Arg103B→His106B
Thr7B→Met10B	

* Routes marked in red involve Met10

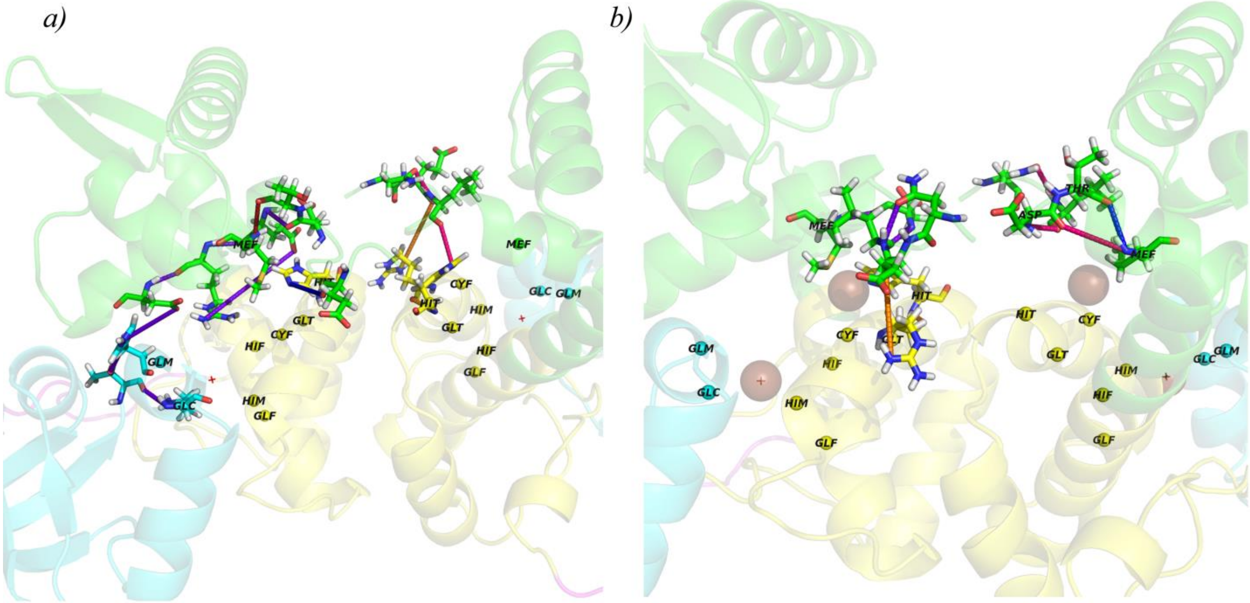

Figure C Iron binding stabilizes the N-terminal residues via a cascade of hydrogen bond connections between the N-terminal residues and MBR. These are illustrated in a) D-nm and b) D-m. In D-m, hydrogen bond formation is observed between Met10 and N-terminal residues which in turn stabilize the N-terminal residues in the metallated system.
