# Supplementary material for: Mechanism of Iron-Dependent Repressor (IdeR) Activation and DNA Binding: A Molecular Dynamics and Protein Structure Network Study
Source: PLoS Comput Biol. 2015 Dec 23;11(12):e1004500. doi: 10.1371/journal.pcbi.1004500 (PMC4689551; doi:10.1371/journal.pcbi.1004500)
Supplement: S3 Text — (PDF) [file pcbi.1004500.s003.pdf]

S3_Text: Supplementary tables

Table A Partial charges on non hydrogen atoms of the metal binding residues before and after *ab-initio* calculations

Residue name	***Atom***	***AMBER charge***	***MS1 charge***		***MS2 charge***
			HIS79	***HIS98***	***HIS106***
HIS	N	-0.42	-0.42	-0.42	-0.42
	CA	0.02	0.02	0.02	0.02
	CB	-0.05	0.10	0.32	-0.22
	CG	-0.03	-0.14	0.08	0.03
	ND1	-0.38	-0.09	-0.23	-0.13
	CE1	0.21	-0.15	-0.15	-0.06
	NE2	-0.57	-0.12	-0.17	-0.08
	CD2	0.13	-0.02	-0.16	-0.13
	C	0.60	0.60	0.60	0.60
	O	-0.57	-0.57	-0.57	-0.57
			GLU83	***GLU172***	***GLU105***
GLU	N	-0.52	-0.52	-0.52	-0.52
	CA	0.04	0.04	0.04	0.04
	CB	0.06	0.25	0.25	-0.10
	CG	0.01	-0.05	-0.05	0.16
	CD	0.81	0.57	0.57	0.56
	OE1	-0.82	-0.62	-0.62	-0.56
	OE2	-0.82	-0.62	-0.62	-0.56
	C	0.54	0.54	0.54	0.54
	O	-0.58	-0.58	-0.58	-0.58
			GLN175		
GLN	N	-0.42	-0.42		
	CA	0.00	0.00		
	CB	0.00	0.00		
	CG	-0.06	0.00		
	CD	0.70	0.22		
	OE1	-0.61	-0.24		
	NE2	-0.94	-0.59		
	C	0.60	0.60		
	O	-0.57	-0.57		

					MET10
MET	N	-0.42			-0.42
	CA	-0.02			-0.02
	CB	0.03			0.04
	CG	0.00			0.04
	SD	-0.27			0.08
	CE	-0.05			-0.05
	C	0.60			0.60
	O	-0.57			-0.57
					CYS102
CYS	N	-0.42			-0.42
	CA	0.04			0.04
	CB	-0.08			-0.18
	SG	-0.11			-0.46
	C	0.60			0.60
	O	-0.57			-0.30
HOH	O	-1			-0.81
FE	FE	2	0.92		0.46

Table B Molecular Dynamics Protocol

Parameters	***MD protocol***
Time step for integration	2fs
Temperature	300K
Temperature coupling	Langevin dynamics
Pressure	1 bar
Electrostatic Interactions	Particle mesh Ewald
Water model	TIP3P
Force Field	ff99SB
Metal ion force field	Modified using MCPB
Size of cubic box	12 Å away from the farthest atom in all directions
Minimization	500 steps [SD] + 2500 steps [CG]
Equilibration	20ps + 50ps + 50ps = 120 ps
Length of production run	100 ns

Table C: List of dynamically stable hydrogen bonds between protein subunits and DNA.

System	*Chain A*			*Chain B*			*Chain C*			*Chain D*		
	DNA	***Protein***	***%O***	***DNA***	***Protein***	***%O***	***DNA***	***Protein***	***%O***	***DNA***	***Protein***	***%O***
FU-nm	10/O2P	28/N	99.3				13'/O2P	60/NH1	71.9	5/O1P	27/NH2	94.7
	10/O1P	27/NH2	98.4				13'/O2P	60/NE	59.1	24'/O2P	37/OG	84.0
	9/O1P	29/NH1	93.9							5/O1P	27/NE	79.7
	9/O2P	29/NH2	90.5							24'/O2P	40/OG1	66.3
	10/O1P	27/NE	75.0							4/O2P	29/NH1	53.5
	17'/O2P	43/NE2	69.4							6/O2P	42/OG	53.3
	18'/O2P	47/NH2	65.6							22'/O2P	47/NH2	53.1
	17'/O2P	47/NH1	62.0									
	11/O2P	27/NH2	53.6									
	17'/O2P	50/NH2	51.8									
FU-m	18'/O2P	7/OG1	62.5	20/O2P	7/OG1	73.5	13'/O1P	27/NH2	99.6	23'/O2P	11/OH	99.7
	17'/O1P	7/N	58.3	21/O2P	37/N	63.0	16/O2P	37/OG	95.1	24'/O2P	37/OG	99.7
	11/O2P	27/NH1	55.1	21/O2P	37/OG	62.5	12'/O1P	29/NH1	81.2	5/O1P	27/NH2	99.7
	17'/O2P	8/OG1	53.6	*[*]20/N4*	*43/OE1*	*56.7*	13'/O1P	27/NE	76.3	22'/O2P	47/NH1	95.4
				20/O2P	36/NE2	56.5	12'/O2P	29/NE	74.2	4/O2P	29/NH2	78.5
				21/O2P	40/OG1	55.4	16/O2P	40/OG1	72.1	4/O1P	29/NH1	78.0
							14/O1P	7/OG1	52.7	24'/O2P	40/OG1	76.6
										[*]23'/N7	*43/NE2*	*71.7*

* Hydrogen bonds involving DNA bases are marked in red and italicized. All other hydrogen bonds are formed between the phosphate group of DNA and protein residue.
%O represents percentage occupancy of the hydrogen bonds.

Table D List of residues forming dynamically stable clusters in the protein-DNA bipartite network

	Chain A	*Chain B*	*Chain C*	*Chain D*
FU-nm	d12 40 d19' 39 d20' 37	60 d8' 27	28 60 d12'	d7 d24' 37 d25' 39 d6
	28 27 d10 60 d9 26		29 d14' 27 28 d13' 60 d12'	36 40 d23'

	d19’ 40 44 43 50 d17’ 47 d18’ ***7***			27 42 d5
	60 d11 27 d10 28 29 d9 26			26 42 d6 27 d5 28 29 d4
				7 37 40 d24’ 36 d23’
FU-m	d19’ 43 d18’ 40	43 d20 40 d21 39 d22 37	d15’ 39 d17 37 d16 40 d15	d7 d22’ 43 d23’ 40 d24’ 37 d25’ 39 d6
	47 d16’ ***8***	36 40 d20 ***2***	26 28 27 d13’ 60 d12’	26 28 27 d5 60 d4
	60 d10 27	***7*** 37 40 d21 36 d20 ***2***	36 40 d15	36 40 d23’
	7 d17’ ***2***	50 d18 47 d19 ***8***	37 40 d16 36 d15 ***7*** d14 47 d13	***2 4 7*** 11 37 39 d24’ 36 d23’
	6 d18’ ***7 8*** 47 d17’***2***	60 d8’ 27	60 d14’ 27 28 d13’ 26 29 d12’	60 d6 27 28 d5 26 29 d4
	60 d10 d11 27			43 47 d22’

* Network are generated at MEC = 2%. Cluster colours represent the different clusters obtained from bipartite network from different DNA entities, blue [nucleobases], green [deoxyribose sugar] and phosphate [orange]. Bold and underlined residues represent the N-terminal region. Also, note that DNA residues have a prefix ‘d’.

Table E Top 20 protein-DNA pairs that contribute maximally to the free energy contribution in FU-nm and FU-m.

FU-nm			FU-m		
DNA	***P***	***E (kcal/mol)***	***DNA***	***P***	***E (kcal/mol)***
**10**	**27A**	**-20.903**	**5**	**27D**	**-21.594**
**13’**	**27C**	**-20.877**	**13’**	**27C**	**-20.080**
**5**	**27D**	**-16.351**	**4**	**29D**	**-13.838**
**17’**	**50A**	**-15.320**	**5**	**29C**	**-12.468**
**17’**	**47A**	**-14.580**	**17’**	**47A**	**-12.240**
**4**	**29D**	**-12.808**	**13’**	**60C**	**-12.227**
**9**	**29A**	**-12.154**	**19**	**47B**	**-11.606**
**7’**	**27B**	**-11.849**	**7’**	**27B**	**-11.179**
**22’**	**47D**	**-11.377**	**14**	**47C**	**-10.754**
**13’**	**60C**	**-10.501**	**15’**	**50A**	**-10.552**
**24’**	**37D**	**-10.339**	**22’**	**47D**	**-10.021**
**8’**	**60B**	**-9.723**	**10**	**60A**	**-9.389**
**11**	**27A**	**-9.130**	**18**	**50B**	**-9.276**
**18’**	**47A**	**-8.848**	**24’**	**37D**	**-8.775**

**12’**	**60C**	**-8.205**	**10**	**27A**	**-8.525**
**12’**	**27A**	**-8.050**	**11**	**27A**	**-8.393**
**11’**	**29C**	**-7.616**	**5**	**60D**	**-8.373**
**6**	**27D**	**-7.362**	**16**	**37C**	**-7.700**
**24’**	**26D**	**-6.972**	**14’**	**60C**	**-6.595**
**24’**	**13D**	**-6.672**	**17’**	**7A**	**-6.563**

*** Pairs are coloured based on protein residue location in the DNA binding domain. Orange represents protein residues from the 2nd helix, blue represents residues from the DBHs and red for N-terminal residues.**

Table F Mean and standard deviation values of the different parameters used to monitor the geometry of the metal center. Values are provided for individual subunit of all the metallated system studied (M1, M2, D-m and FU). Standard deviation is calculated over all the snapshots. The distance values are compared to experimental values obtained from (Feese *et.al.* J Biol Chem. 2001;276(8):5959-66 and Wisedchaisri G *et.al.* Journal of molecular biology. 2004;342(4):1155-69), representing the crystal structures IFX7 and 1U8R. Mean and standard deviation of these values are obtained from individual monomeric subunits of the crystal structure as provided in the literature. The angles are compared to expected values (text book) of the given geometry (distorted trigonal bipyramidal geometry for MS1 and octahedral geometry for MS2).

**Parameter**	**M1**	**M2**	**Dm-A**	**Dm-B**	**FU-A**	**FU-B**	**FU-C**	**FU-D**	**Known**
Distance between Fe and coordinating ligands									
Fe-79@NE2	2.05±0.06	2.07±0.06	2.08±0.06	2.05±0.06	2.08±0.06	2.05±0.06	2.07±0.06	2.07±0.06	2.05±0.10
Fe-83@OE2	1.99±0.06	1.99±0.06	1.98±0.06	1.99±0.06	1.98±0.06	1.99±0.06	1.99±0.06	1.98±0.06	1.99±0.10
Fe-98@ND1	2.06±0.06	2.07±0.05	2.07±0.06	2.06±0.06	2.07±0.06	2.06±0.06	2.06±0.06	2.06±0.06	2.07±0.09
Fe-172@OE1	2.05±0.06	2.04±0.06	2.01±0.07	2.04±0.06	2.00±0.06	2.02±0.06	2.03±0.06	1.99±0.06	1.92±0.11
Fe-175@OE1	2.02±0.06	2.03±0.06	2.00±0.06	2.01±0.07	2.01±0.06	2.01±0.06	2.00±0.06	2.01±0.07	1.94±0.14
Fe-10@SD	NA	2.37±0.06	2.39±0.07	2.39±0.06	2.39±0.07	2.37±0.06	2.39±0.06	2.39±0.06	2.32±0.11
Fe-102@O	NA	1.83±0.06	1.83±0.06	1.83±0.06	1.83±0.06	1.83±0.06	1.84±0.06	1.84±0.06	2.06±0.13
Fe-102@SG	NA	2.19±0.07	2.18±0.07	2.19±0.07	2.18±0.07	2.19±0.06	2.19±0.07	2.18±0.07	2.88±0.30
Fe-105@OE1	NA	1.98±0.06	1.96±0.06	1.97±0.06	1.96±0.06	1.96±0.06	1.97±0.06	1.97±0.06	1.97±0.08
Fe-106@NE2	NA	2.13±0.06	2.14±0.06	2.14±0.06	2.15±0.06	2.14±0.06	2.14±0.06	2.13±0.06	2.13±0.07
Angle between Fe and two coordinating ligands									
98@ND1-Fe-172@OE1	89.3±2.49	89.4±2.53	87.0±3.07	88.5±2.69	85.8±2.61	88.5±2.66	89.2±2.84	89.2±2.84	~90
172@OE1-Fe-79@NE2	87.0±2.96	88.9±3.13	92.2±3.47	88.6±3.39	92.7±3.30	87.8±3.23	89.1±3.33	92.2±3.02	~90

79@NE2-Fe-83@OE2	89.5±2.58	90.1±2.76	91.6±2.73	90.3±2.80	92.1±2.53	90.3±2.74	90.9±2.78	91.8±2.61	~90
83@OE2-Fe-175@OE1	90.4±2.93	89.2±2.94	90.3±2.90	90.2±2.99	90.4±2.89	90.4±2.98	90.0±2.89	90.2±2.94	~90
175@OE1-Fe-98@ND1	130.5±2.3	130.9±2.2	131.2±2.4	130.6±2.3	131.6±2.3	130.1±2.2	130.3±2.3	131.4±2.3	~120
105@OE2-Fe-106@NE2	NA	76.8±2.52	78.0±3.88	76.5±2.57	76.6±2.79	76.4±2.59	76.3±2.55	76.6±2.62	~90
106@NE2-Fe-102@O	NA	80.6±2.49	80.9±2.54	80.6±2.43	80.2±2.44	80.1±2.40	79.8±2.47	80.7±2.49	~90
102@O-Fe-102@SG	NA	107.3±2.6	107.6±2.8	107.0±2.6	106.8±2.6	107.4±2.5	107.2±2.6	107.1±2.8	~90

Table G

Coordinates of the metal centers used to generate the electrostatic parameters using MCPB.

Metal Site1 sidechain model for force constants:

```
REMARK
ATOM    627  CB  HIF     1       6.261  41.034   3.476
ATOM    628  CG  HIF     1       5.188  41.773   2.727
ATOM    629  ND1 HIF     1       4.801  43.044   3.085
ATOM    630  CD2 HIF     1       4.441  41.427   1.654
ATOM    631  CE1 HIF     1       3.847  43.454   2.266
ATOM    632  NE2 HIF     1       3.621  42.494   1.389
ATOM   2415  HB2 HIF     1       7.021  41.755   3.778
ATOM   2416  HB3 HIF     1       6.703  40.302   2.800
ATOM   2417  HD1 HIF     1       5.178  43.586   3.849
ATOM   2418  HE1 HIF     1       3.402  44.440   2.398
ATOM   2419  HD2 HIF     1       4.390  40.533   1.032
ATOM    661  CB  GLF     2       0.120  38.748   2.987
ATOM    662  CG  GLF     2       0.586  40.176   2.719
ATOM    663  CD  GLF     2       1.075  40.440   1.316
ATOM    664  OE1 GLF     2       1.603  39.502   0.685
ATOM    665  OE2 GLF     2       0.964  41.577   0.806
ATOM   2451  HB2 GLF     2       0.947  38.072   2.771
ATOM   2452  HB3 GLF     2      -0.712  38.528   2.319
ATOM   2453  HG2 GLF     2      -0.239  40.867   2.895
ATOM   2454  HG3 GLF     2       1.413  40.420   3.386
ATOM    785  CB  HIM     3      -0.523  44.821   0.179
ATOM    786  CG  HIM     3       0.820  45.444  -0.116
ATOM    787  ND1 HIM     3       1.967  44.704  -0.160
ATOM    788  CD2 HIM     3       1.155  46.741  -0.348
ATOM    789  CE1 HIM     3       2.985  45.518  -0.417
ATOM    790  NE2 HIM     3       2.512  46.756  -0.539
ATOM   2577  HB2 HIM     3      -0.624  43.888  -0.375
ATOM   2578  HB3 HIM     3      -1.318  45.506  -0.115
```

```
ATOM   2579  HE1 HIM     3        3.997  45.119  -0.492
ATOM   2580  HE2 HIM     3        3.071  47.573  -0.741
ATOM   2581  HD2 HIM     3        0.587  47.670  -0.402
ATOM   1340  CB  GLM     4        5.396  44.105  -3.202
ATOM   1341  CG  GLM     4        5.831  42.753  -2.638
ATOM   1342  CD  GLM     4        4.678  41.969  -2.022
ATOM   1343  OE1 GLM     4        3.643  42.572  -1.668
ATOM   1344  OE2 GLM     4        4.767  40.735  -1.923
ATOM   3134  HB2 GLM     4        6.273  44.585  -3.637
ATOM   3135  HB3 GLM     4        5.024  44.709  -2.375
ATOM   3136  HG2 GLM     4        6.256  42.145  -3.437
ATOM   3137  HG3 GLM     4        6.580  42.907  -1.861
ATOM   1366  CB  GLC     5        0.188  44.691  -3.464
ATOM   1367  CG  GLC     5        0.083  43.242  -3.885
ATOM   1368  CD  GLC     5        0.852  42.315  -2.959
ATOM   1369  OE1 GLC     5        0.810  42.457  -1.747
ATOM   1370  NE2 GLC     5        1.565  41.359  -3.532
ATOM   3156  HB2 GLC     5        1.250  44.888  -3.315
ATOM   3157  HB3 GLC     5       -0.333  44.769  -2.510
ATOM   3158  HG2 GLC     5       -0.963  42.935  -3.876
ATOM   3159  HG3 GLC     5        0.487  43.125  -4.891
ATOM   3160 HE21 GLC     5        2.096  40.717  -2.960
ATOM   3161 HE22 GLC     5        1.575  41.275  -4.538
ATOM   1779 FE   FE1     6        2.243  42.623  -0.240
ATOM     54  C   CH3     7        5.687  40.325   4.716
ATOM     55  H1  CH3     7        6.484  39.801   5.236
ATOM     56  H2  CH3     7        4.928  39.613   4.408
ATOM     57  H3  CH3     7        5.244  41.060   5.382
ATOM     58  C   CH3     8       -0.327  38.573   4.450
ATOM     59  H1  CH3     8       -0.652  37.550   4.610
ATOM     60  H2  CH3     8       -1.148  39.250   4.661
ATOM     61  H3  CH3     8        0.504  38.796   5.112
ATOM     62  C   CH3     9       -0.677  44.514   1.680
ATOM     63  H1  CH3     9       -1.651  44.068   1.861
ATOM     64  H2  CH3     9       -0.590  45.434   2.249
ATOM     65  H3  CH3     9        0.101  43.823   1.991
ATOM     66  C   CH3    10        4.300  43.936  -4.270
ATOM     67  H1  CH3    10        4.012  44.912  -4.651
ATOM     68  H2  CH3    10        3.435  43.452  -3.828
ATOM     69  H3  CH3    10        4.678  43.327  -5.085
ATOM     70  C   CH3    11       -0.422  45.625  -4.525
ATOM     71  H1  CH3    11       -0.332  46.656  -4.196
ATOM     72  H2  CH3    11       -1.470  45.380  -4.664
ATOM     73  H3  CH3    11        0.106  45.499  -5.466
```

Metal Site1 large model for RESP charges:

```
REMARK
ATOM    615  CH3 ACE     1        7.776  38.196   7.083
ATOM    616  C   ACE     1        6.562  38.865   6.446
```

ATOM	617	O	ACE	1	5.436	38.697	6.905
ATOM	3582	HH31	ACE	1	7.483	37.580	7.933
ATOM	3583	HH32	ACE	1	8.264	37.574	6.333
ATOM	3584	HH33	ACE	1	8.466	38.971	7.416
ATOM	623	N	HIF	2	6.831	39.649	5.384
ATOM	624	CA	HIF	2	5.710	40.317	4.720
ATOM	625	C	HIF	2	4.658	39.317	4.259
ATOM	626	O	HIF	2	3.458	39.467	4.490
ATOM	627	CB	HIF	2	6.261	41.034	3.476
ATOM	628	CG	HIF	2	5.188	41.773	2.727
ATOM	629	ND1	HIF	2	4.801	43.044	3.085
ATOM	630	CD2	HIF	2	4.441	41.427	1.654
ATOM	631	CE1	HIF	2	3.847	43.454	2.266
ATOM	632	NE2	HIF	2	3.621	42.494	1.389
ATOM	2413	H	HIF	2	7.785	39.760	5.071
ATOM	2414	HA	HIF	2	5.241	41.016	5.413
ATOM	2415	HB2	HIF	2	7.021	41.755	3.778
ATOM	2416	HB3	HIF	2	6.703	40.302	2.800
ATOM	2417	HD1	HIF	2	5.178	43.586	3.849
ATOM	2418	HE1	HIF	2	3.402	44.440	2.398
ATOM	2419	HD2	HIF	2	4.390	40.533	1.032
ATOM	633	N	NME	3	5.133	38.287	3.539
ATOM	634	CH3	NME	3	4.207	37.337	2.913
ATOM	3585	H	NME	3	6.130	38.168	3.430
ATOM	3586	HH31	NME	3	4.354	37.344	1.833
ATOM	3587	HH32	NME	3	4.397	36.335	3.299
ATOM	3588	HH33	NME	3	3.181	37.625	3.141
ATOM	653	CH3	ACE	4	1.836	38.348	7.562
ATOM	654	C	ACE	4	0.588	38.471	6.689
ATOM	655	O	ACE	4	-0.549	38.374	7.178
ATOM	3589	HH31	ACE	4	1.572	38.285	8.618
ATOM	3590	HH32	ACE	4	2.379	37.450	7.268
ATOM	3591	HH33	ACE	4	2.465	39.223	7.396
ATOM	657	N	GLF	5	0.799	38.556	5.367
ATOM	658	CA	GLF	5	-0.328	38.561	4.441
ATOM	659	C	GLF	5	-1.171	37.293	4.557
ATOM	660	O	GLF	5	-2.398	37.412	4.566
ATOM	661	CB	GLF	5	0.120	38.748	2.987
ATOM	662	CG	GLF	5	0.586	40.176	2.719
ATOM	663	CD	GLF	5	1.075	40.440	1.316
ATOM	664	OE1	GLF	5	1.603	39.502	0.685
ATOM	665	OE2	GLF	5	0.964	41.577	0.806
ATOM	2449	H	GLF	5	1.743	38.617	5.012
ATOM	2450	HA	GLF	5	-0.970	39.416	4.654
ATOM	2451	HB2	GLF	5	0.947	38.072	2.771
ATOM	2452	HB3	GLF	5	-0.712	38.528	2.319
ATOM	2453	HG2	GLF	5	-0.239	40.867	2.895
ATOM	2454	HG3	GLF	5	1.413	40.420	3.386
ATOM	666	N	NME	6	-0.539	36.124	4.637
ATOM	667	CH3	NME	6	-1.273	34.862	4.794
ATOM	3592	H	NME	6	0.469	36.094	4.590
ATOM	3593	HH31	NME	6	-1.056	34.208	3.950

```
ATOM   3594 HH32 NME      6       -0.965  34.375   5.719
ATOM   3595 HH33 NME      6       -2.343  35.066   4.830
ATOM    775  CH3 ACE      7       -3.620  43.182   3.653
ATOM    776  C   ACE      7       -2.223  43.694   3.299
ATOM    777  O   ACE      7       -1.350  43.680   4.160
ATOM   3596 HH31 ACE      7       -3.681  42.902   4.705
ATOM   3597 HH32 ACE      7       -4.343  43.970   3.444
ATOM   3598 HH33 ACE      7       -3.840  42.314   3.032
ATOM    781  N   HIM      8       -1.966  44.082   2.052
ATOM    782  CA  HIM      8       -0.623  44.536   1.689
ATOM    783  C   HIM      8       -0.178  45.731   2.516
ATOM    784  O   HIM      8        0.967  45.699   3.027
ATOM    785  CB  HIM      8       -0.523  44.821   0.179
ATOM    786  CG  HIM      8        0.820  45.444  -0.116
ATOM    787  ND1 HIM      8        1.967  44.704  -0.160
ATOM    788  CD2 HIM      8        1.155  46.741  -0.348
ATOM    789  CE1 HIM      8        2.985  45.518  -0.417
ATOM    790  NE2 HIM      8        2.512  46.756  -0.539
ATOM   2575  H   HIM      8       -2.700  44.062   1.359
ATOM   2576  HA  HIM      8        0.090  43.728   1.851
ATOM   2577  HB2 HIM      8       -0.624  43.888  -0.375
ATOM   2578  HB3 HIM      8       -1.318  45.506  -0.115
ATOM   2579  HE1 HIM      8        3.997  45.119  -0.492
ATOM   2580  HE2 HIM      8        3.071  47.573  -0.741
ATOM   2581  HD2 HIM      8        0.587  47.670  -0.402
ATOM    791  N   NME      9       -0.986  46.782   2.660
ATOM    792  CH3 NME      9       -0.511  47.937   3.432
ATOM   3599  H   NME      9       -1.905  46.770   2.241
ATOM   3600 HH31 NME      9       -0.503  48.822   2.796
ATOM   3601 HH32 NME      9       -1.175  48.106   4.280
ATOM   3602 HH33 NME      9        0.498  47.742   3.794
ATOM   1330  CH3 ACE     10        4.063  41.378  -7.077
ATOM   1331  C   ACE     10        3.759  42.597  -6.227
ATOM   1332  O   ACE     10        2.684  43.163  -6.353
ATOM   3603 HH31 ACE     10        3.288  41.219  -7.826
ATOM   3604 HH32 ACE     10        5.022  41.529  -7.572
ATOM   3605 HH33 ACE     10        4.124  40.506  -6.426
ATOM   1336  N   GLM     11        4.614  42.945  -5.247
ATOM   1337  CA  GLM     11        4.294  43.971  -4.279
ATOM   1338  C   GLM     11        3.974  45.337  -4.876
ATOM   1339  O   GLM     11        3.144  46.053  -4.317
ATOM   1340  CB  GLM     11        5.396  44.105  -3.202
ATOM   1341  CG  GLM     11        5.831  42.753  -2.638
ATOM   1342  CD  GLM     11        4.678  41.969  -2.022
ATOM   1343  OE1 GLM     11        3.643  42.572  -1.668
ATOM   1344  OE2 GLM     11        4.767  40.735  -1.923
ATOM   3132  H   GLM     11        5.509  42.483  -5.175
ATOM   3133  HA  GLM     11        3.420  43.668  -3.703
ATOM   3134  HB2 GLM     11        6.273  44.585  -3.637
ATOM   3135  HB3 GLM     11        5.024  44.709  -2.375
ATOM   3136  HG2 GLM     11        6.256  42.145  -3.437
ATOM   3137  HG3 GLM     11        6.580  42.907  -1.861
```

```
ATOM   1345  N   NME    12        4.609  45.735  -5.969
ATOM   1346  CH3 NME    12        4.352  47.043  -6.564
ATOM   3606  H   NME    12        5.287  45.126  -6.406
ATOM   3607 HH31 NME    12        5.274  47.624  -6.577
ATOM   3608 HH32 NME    12        3.990  46.914  -7.584
ATOM   3609 HH33 NME    12        3.600  47.569  -5.976
ATOM   1356  CH3 ACE    13        0.817  46.499  -7.958
ATOM   1357  C   ACE    13       -0.077  46.485  -6.742
ATOM   1358  O   ACE    13       -1.041  47.245  -6.620
ATOM   3610 HH31 ACE    13        0.390  47.112  -8.752
ATOM   3611 HH32 ACE    13        1.789  46.900  -7.671
ATOM   3612 HH33 ACE    13        0.937  45.476  -8.314
ATOM   1362  N   GLC    14        0.350  45.759  -5.699
ATOM   1363  CA  GLC    14       -0.392  45.719  -4.452
ATOM   1364  C   GLC    14       -0.340  47.079  -3.763
ATOM   1365  O   GLC    14       -1.279  47.388  -3.038
ATOM   1366  CB  GLC    14        0.188  44.691  -3.464
ATOM   1367  CG  GLC    14        0.083  43.242  -3.885
ATOM   1368  CD  GLC    14        0.852  42.315  -2.959
ATOM   1369  OE1 GLC    14        0.810  42.457  -1.747
ATOM   1370  NE2 GLC    14        1.565  41.359  -3.532
ATOM   3154  H   GLC    14        1.205  45.226  -5.775
ATOM   3155  HA  GLC    14       -1.432  45.462  -4.655
ATOM   3156  HB2 GLC    14        1.250  44.888  -3.315
ATOM   3157  HB3 GLC    14       -0.333  44.769  -2.510
ATOM   3158  HG2 GLC    14       -0.963  42.935  -3.876
ATOM   3159  HG3 GLC    14        0.487  43.125  -4.891
ATOM   3160 HE21 GLC    14        2.096  40.717  -2.960
ATOM   3161 HE22 GLC    14        1.575  41.275  -4.538
ATOM   1371  N   NME    15        0.717  47.853  -3.960
ATOM   1372  CH3 NME    15        0.916  49.144  -3.312
ATOM   3613  H   NME    15        1.446  47.556  -4.593
ATOM   3614 HH31 NME    15        1.832  49.118  -2.722
ATOM   3615 HH32 NME    15        0.995  49.923  -4.070
ATOM   3616 HH33 NME    15        0.070  49.357  -2.659
ATOM   1779 FE   FE1    16        2.243  42.623  -0.240
```

Metal Site 2 sidechain model for force constants:

```
REMARK
ATOM     76  CB  MEF     1       11.177  50.490   3.310
ATOM     77  CG  MEF     1        9.946  50.055   4.111
ATOM     78  SD  MEF     1        9.147  48.576   3.427
ATOM     79  CE  MEF     1        8.367  49.275   1.970
ATOM   1847  HB2 MEF     1       11.717  51.243   3.884
ATOM   1848  HB3 MEF     1       10.843  50.924   2.368
ATOM   1849  HG2 MEF     1       10.240  49.830   5.136
ATOM   1850  HG3 MEF     1        9.210  50.859   4.113
ATOM   1851  HE1 MEF     1        8.752  48.781   1.078
ATOM   1852  HE2 MEF     1        7.289  49.128   2.029
ATOM   1853  HE3 MEF     1        8.586  50.342   1.917
ATOM    811  CA  CYF     2        4.124  46.459   4.853
```

ATOM	2599	HA	CYF	2	4.923	45.846	4.435
ATOM	812	C	CYF	2	4.623	47.092	6.162
ATOM	813	O	CYF	2	5.797	47.442	6.269
ATOM	814	CB	CYF	2	3.929	47.575	3.825
ATOM	2600	HB2	CYF	2	3.407	47.180	2.954
ATOM	2601	HB3	CYF	2	3.340	48.378	4.267
ATOM	815	SG	CYF	2	5.511	48.287	3.256
ATOM	845	CB	GLT	3	7.257	43.149	6.644
ATOM	846	CG	GLT	3	7.641	44.601	6.305
ATOM	847	CD	GLT	3	7.529	44.885	4.806
ATOM	848	OE1	GLT	3	7.312	43.942	4.036
ATOM	849	OE2	GLT	3	7.629	46.052	4.355
ATOM	2627	HB2	GLT	3	8.060	42.497	6.301
ATOM	2628	HB3	GLT	3	6.339	42.907	6.108
ATOM	2629	HG2	GLT	3	6.977	45.286	6.833
ATOM	2630	HG3	GLT	3	8.671	44.786	6.611
ATOM	854	CB	HIT	4	9.345	46.755	9.979
ATOM	855	CG	HIT	4	9.257	47.092	8.514
ATOM	856	ND1	HIT	4	10.238	46.778	7.618
ATOM	857	CD2	HIT	4	8.273	47.704	7.817
ATOM	858	CE1	HIT	4	9.881	47.183	6.402
ATOM	859	NE2	HIT	4	8.692	47.759	6.498
ATOM	2633	HB2	HIT	4	8.495	47.200	10.496
ATOM	2634	HB3	HIT	4	10.270	47.167	10.382
ATOM	2635	HD1	HIT	4	11.108	46.310	7.830
ATOM	2636	HE1	HIT	4	10.543	47.010	5.554
ATOM	2637	HD2	HIT	4	7.305	48.117	8.099
ATOM	6963	FE	FE2	5	7.280	47.921	4.876
ATOM	7031	O	OH1	6	6.986	50.124	5.514
ATOM	3570	H1	OH1	6	6.135	50.147	5.951
ATOM	44	C	CH3	7	12.107	49.296	3.025
ATOM	45	H1	CH3	7	12.968	49.635	2.457
ATOM	46	H2	CH3	7	11.569	48.547	2.453
ATOM	47	H3	CH3	7	12.440	48.864	3.964
ATOM	48	C	CH3	8	7.044	42.968	8.158
ATOM	49	H1	CH3	8	6.775	41.937	8.366
ATOM	50	H2	CH3	8	6.247	43.624	8.493
ATOM	51	H3	CH3	8	7.961	43.216	8.685
ATOM	52	C	CH3	9	9.331	45.231	10.202
ATOM	53	H1	CH3	9	9.396	45.020	11.265
ATOM	54	H2	CH3	9	10.178	44.783	9.693
ATOM	55	H3	CH3	9	8.410	44.815	9.806
ATOM	56	C	CH3	10	2.834	45.638	5.036
ATOM	57	H1	CH3	10	3.003	44.856	5.770
ATOM	58	H2	CH3	10	2.552	45.191	4.089
ATOM	59	H3	CH3	10	2.036	46.290	5.379
ATOM	60	C	CH3	11	3.479	47.237	7.182
ATOM	61	H1	CH3	11	3.861	47.686	8.094
ATOM	62	H2	CH3	11	3.067	46.258	7.406
ATOM	63	H3	CH3	11	2.700	47.870	6.767

Metal Site2 large model for RESP charges:

```
REMARK
ATOM     64  CH3 ACE     1      14.361  47.494   5.544
ATOM     65  C   ACE     1      13.495  47.805   4.335
ATOM     66  O   ACE     1      13.500  46.974   3.419
ATOM   3581 HH31 ACE     1      14.771  46.486   5.484
ATOM   3582 HH32 ACE     1      15.176  48.216   5.583
ATOM   3583 HH33 ACE     1      13.751  47.586   6.443
ATOM     72  N   MEF     2      12.901  48.990   4.218
ATOM     73  CA  MEF     2      12.104  49.299   3.026
ATOM     74  C   MEF     2      12.910  49.485   1.762
ATOM     75  O   MEF     2      12.625  49.106   0.627
ATOM     76  CB  MEF     2      11.177  50.490   3.310
ATOM     77  CG  MEF     2       9.946  50.055   4.111
ATOM     78  SD  MEF     2       9.147  48.576   3.427
ATOM     79  CE  MEF     2       8.367  49.275   1.970
ATOM   1845  H   MEF     2      13.001  49.676   4.952
ATOM   1846  HA  MEF     2      11.403  48.486   2.837
ATOM   1847  HB2 MEF     2      11.717  51.243   3.884
ATOM   1848  HB3 MEF     2      10.843  50.924   2.368
ATOM   1849  HG2 MEF     2      10.240  49.830   5.136
ATOM   1850  HG3 MEF     2       9.210  50.859   4.113
ATOM   1851  HE1 MEF     2       8.752  48.781   1.078
ATOM   1852  HE2 MEF     2       7.289  49.128   2.029
ATOM   1853  HE3 MEF     2       8.586  50.342   1.917
ATOM     80  N   NME     3      14.159  49.967   1.956
ATOM     81  CH3 NME     3      15.179  50.159   0.934
ATOM   3584  H   NME     3      14.456  50.232   2.884
ATOM   3585 HH31 NME     3      15.460  51.211   0.893
ATOM   3586 HH32 NME     3      16.056  49.559   1.178
ATOM   3587 HH33 NME     3      14.786  49.850  -0.035
ATOM    806  CH3 ACE     4       1.772  43.705   6.099
ATOM    807  C   ACE     4       3.026  44.516   5.828
ATOM    808  O   ACE     4       4.097  44.172   6.343
ATOM   3588 HH31 ACE     4       1.994  42.831   6.712
ATOM   3589 HH32 ACE     4       1.052  44.339   6.617
ATOM   3590 HH33 ACE     4       1.352  43.383   5.146
ATOM    810  N   CYF     5       2.946  45.630   5.088
ATOM    811  CA  CYF     5       4.124  46.459   4.853
ATOM    812  C   CYF     5       4.623  47.092   6.162
ATOM    813  O   CYF     5       5.797  47.442   6.269
ATOM    814  CB  CYF     5       3.929  47.575   3.825
ATOM    815  SG  CYF     5       5.511  48.287   3.256
ATOM   2598  H   CYF     5       2.061  45.903   4.686
ATOM   2599  HA  CYF     5       4.923  45.846   4.435
ATOM   2600  HB2 CYF     5       3.407  47.180   2.954
ATOM   2601  HB3 CYF     5       3.340  48.378   4.267
ATOM    816  N   NME     6       3.750  47.279   7.133
ATOM    817  CH3 NME     6       4.114  47.706   8.467
ATOM   3591  H   NME     6       2.767  47.122   6.961
ATOM   3592 HH31 NME     6       3.620  48.651   8.694
```

ATOM	3593	HH32	NME	6	3.802	46.951	9.189
ATOM	3594	HH33	NME	6	5.194	47.838	8.525
ATOM	828	CH3	ACE	7	4.048	44.238	10.128
ATOM	829	C	ACE	7	5.363	43.553	9.821
ATOM	830	O	ACE	7	6.046	43.050	10.732
ATOM	3595	HH31	ACE	7	3.788	44.134	11.181
ATOM	3596	HH32	ACE	7	4.139	45.295	9.878
ATOM	3597	HH33	ACE	7	3.268	43.786	9.514
ATOM	841	N	GLT	8	5.795	43.609	8.555
ATOM	842	CA	GLT	8	7.037	42.942	8.166
ATOM	843	C	GLT	8	8.240	43.444	8.935
ATOM	844	O	GLT	8	9.264	42.762	8.952
ATOM	845	CB	GLT	8	7.257	43.149	6.644
ATOM	846	CG	GLT	8	7.641	44.601	6.305
ATOM	847	CD	GLT	8	7.529	44.885	4.806
ATOM	848	OE1	GLT	8	7.312	43.942	4.036
ATOM	849	OE2	GLT	8	7.629	46.052	4.355
ATOM	2625	H	GLT	8	5.260	44.115	7.864
ATOM	2626	HA	GLT	8	6.948	41.871	8.350
ATOM	2627	HB2	GLT	8	8.060	42.497	6.301
ATOM	2628	HB3	GLT	8	6.339	42.907	6.108
ATOM	2629	HG2	GLT	8	6.977	45.286	6.833
ATOM	2630	HG3	GLT	8	8.671	44.786	6.611
ATOM	850	N	HIT	9	8.185	44.666	9.490
ATOM	851	CA	HIT	9	9.330	45.220	10.185
ATOM	852	C	HIT	9	9.373	44.928	11.676
ATOM	853	O	HIT	9	10.336	45.355	12.342
ATOM	854	CB	HIT	9	9.345	46.755	9.979
ATOM	855	CG	HIT	9	9.257	47.092	8.514
ATOM	856	ND1	HIT	9	10.238	46.778	7.618
ATOM	857	CD2	HIT	9	8.273	47.704	7.817
ATOM	858	CE1	HIT	9	9.881	47.183	6.402
ATOM	859	NE2	HIT	9	8.692	47.759	6.498
ATOM	2631	H	HIT	9	7.336	45.209	9.424
ATOM	2632	HA	HIT	9	10.247	44.812	9.759
ATOM	2633	HB2	HIT	9	8.495	47.200	10.496
ATOM	2634	HB3	HIT	9	10.270	47.167	10.382
ATOM	2635	HD1	HIT	9	11.108	46.310	7.830
ATOM	2636	HE1	HIT	9	10.543	47.010	5.554
ATOM	2637	HD2	HIT	9	7.305	48.117	8.099
ATOM	860	N	NME	10	8.315	44.344	12.229
ATOM	861	CH3	NME	10	8.275	44.116	13.671
ATOM	3598	H	NME	10	7.539	44.058	11.649
ATOM	3599	HH31	NME	10	7.422	44.643	14.099
ATOM	3600	HH32	NME	10	8.178	43.048	13.868
ATOM	3601	HH33	NME	10	9.195	44.486	14.124
ATOM	6963	FE	FE2	11	7.280	47.921	4.876
ATOM	7031	O	OH1	12	6.986	50.124	5.514
ATOM	3570	H1	OH1	12	6.549	50.789	4.981
