# Supplementary figures and images for: Mechanism of Iron-Dependent Repressor (IdeR) Activation and DNA Binding: A Molecular Dynamics and Protein Structure Network Study
Source: PLoS Comput Biol. 2015 Dec 23;11(12):e1004500. doi: 10.1371/journal.pcbi.1004500 (PMC4689551; doi:10.1371/journal.pcbi.1004500)

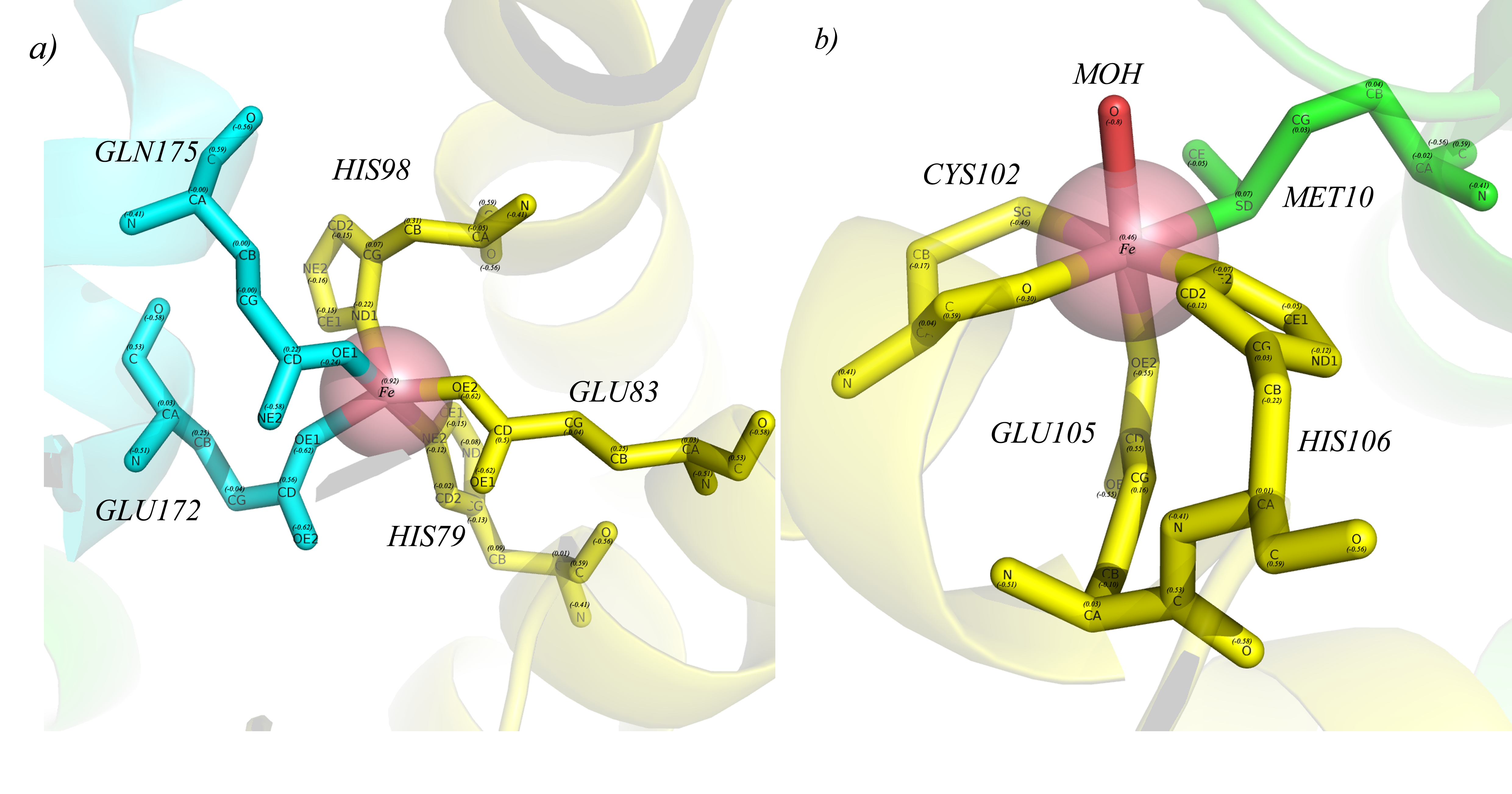

Supplement: S1 Fig — MS1 forms a distorted bipyramidal geometry using sidechain atoms of five residues, while MS2 forms an octahedral geometry using sidechain atoms of four residues and a water molecule. In this case, Cys102 provides two ligands to the metal ion. Each residue is colour coded based on the domain, to which they belong, [green: DBD, yellow: DD, pink: linker and cyan: SH3 like domain]. (TIF) [file pcbi.1004500.s004.tif]

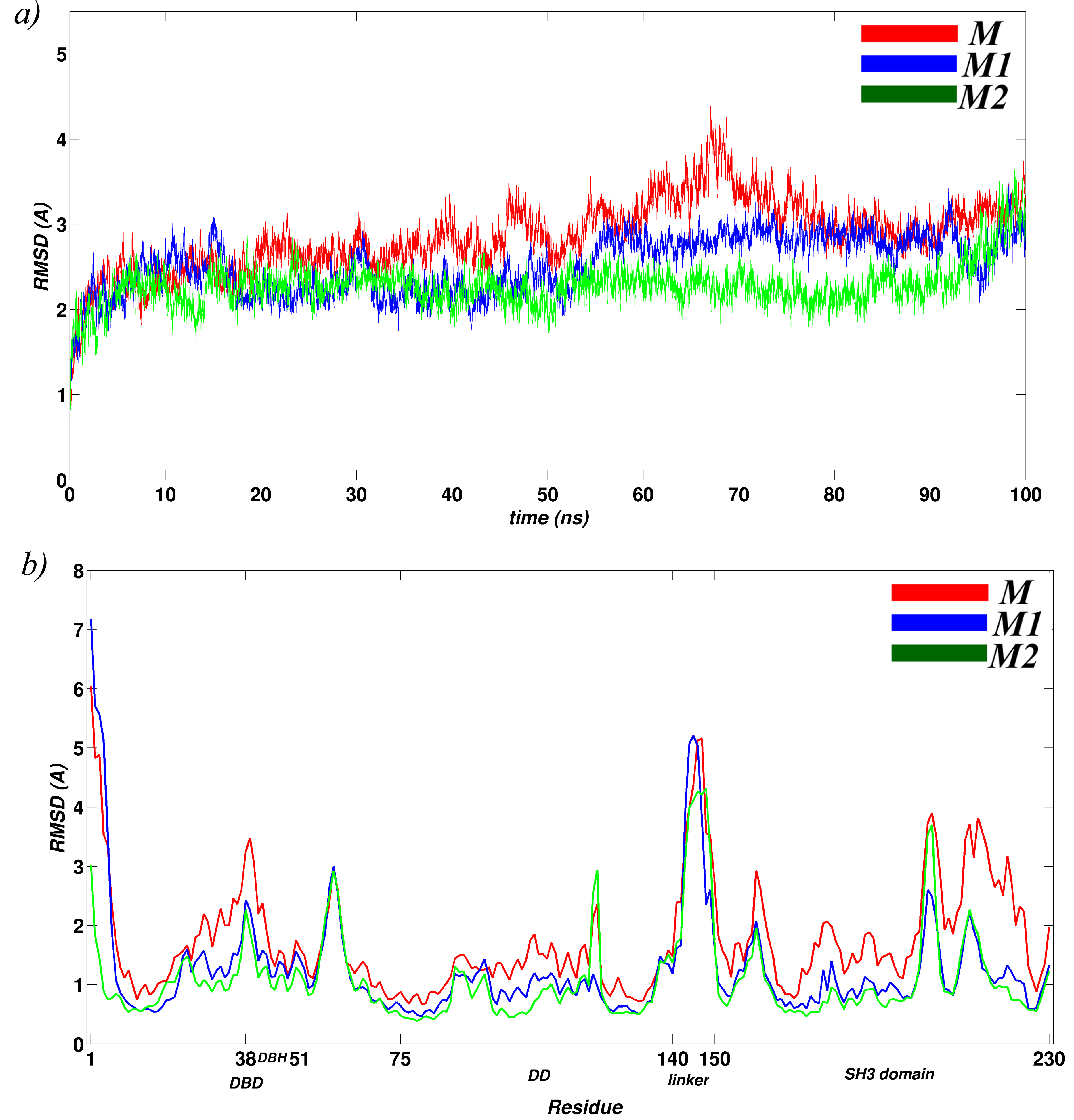

Supplement: S2 Fig — a) Cα RMSD profile of the three monomeric systems, M, M1 and M2 for 100 ns simulations b) Residue wise fluctuation for each system. Residue numbers are also mapped to individual domains. (TIF) [file pcbi.1004500.s005.tif]

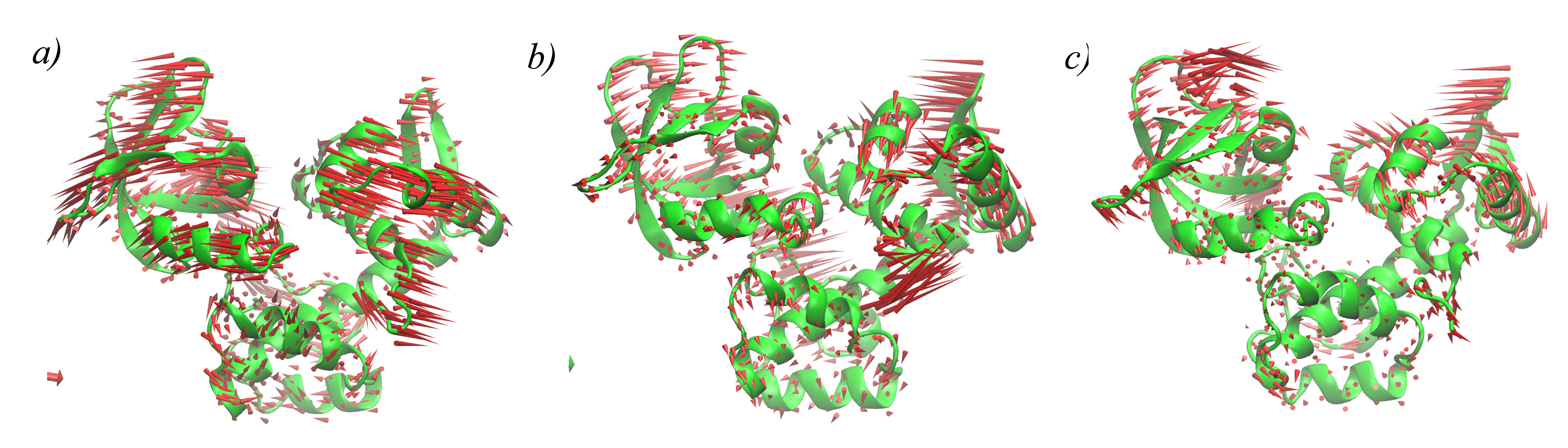

Supplement: S3 Fig — Eigen vectors corresponding to the first eigen value are projected on a) no iron bound form [M], b) Fe bound at MS1 [M1] and c) iron bound at both MS1 and MS2 [M2]. The red arrows indicate the direction as well as the magnitude of motions in the systems. Based on the domain movements, the structures are termed as the ‘open’ and the ‘closed’ conformations. (TIF) [file pcbi.1004500.s006.tif]

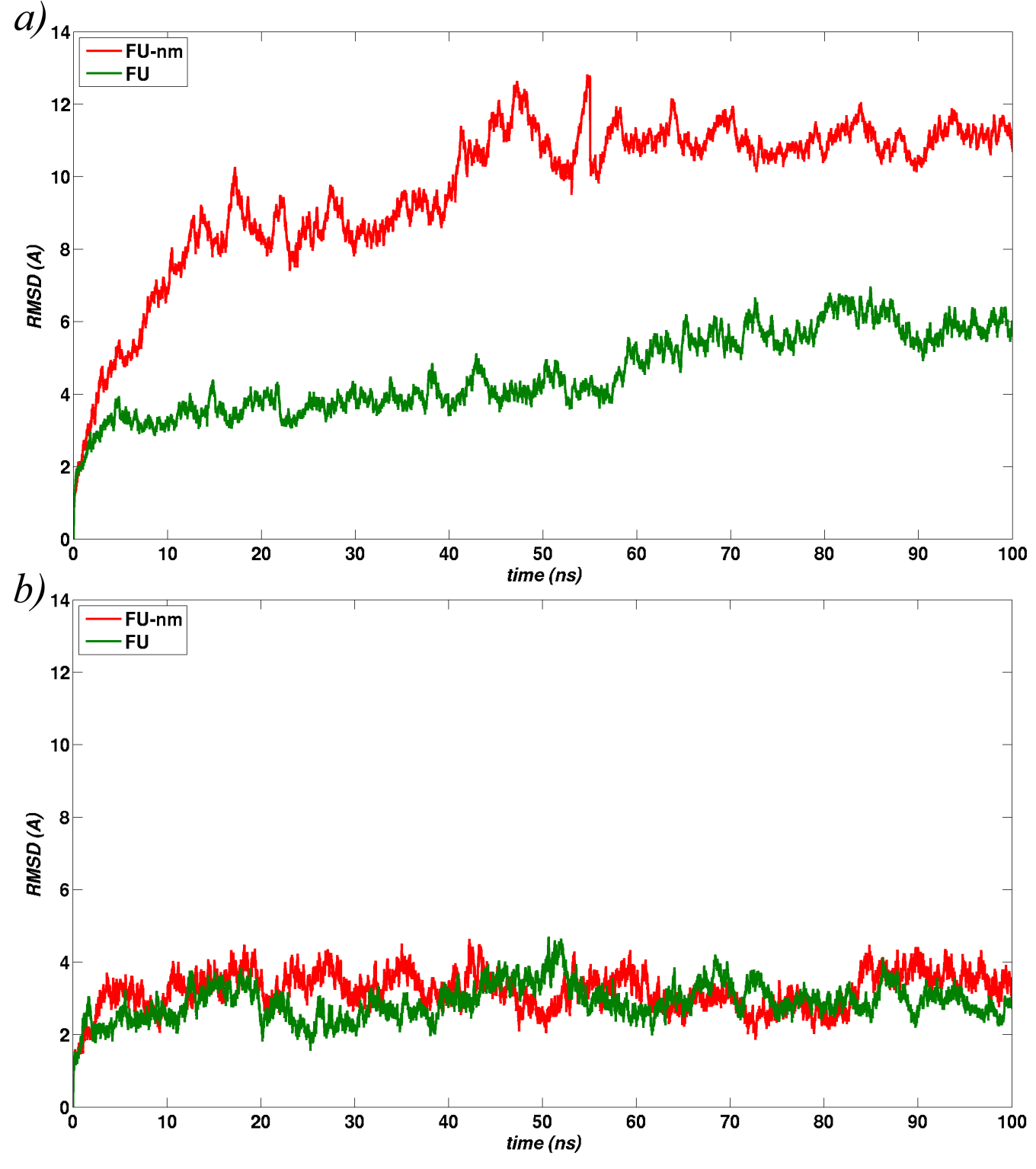

Supplement: S4 Fig — a) Cαbackbone RMSD of the protein subunits of the FU-nm/m cases are plotted over the 100 ns simulations The large variation observed for FU-nm is discussed in details in the text. b) All atom RMSD of the DNA subunit is plotted as a function of time. (TIF) [file pcbi.1004500.s007.tif]

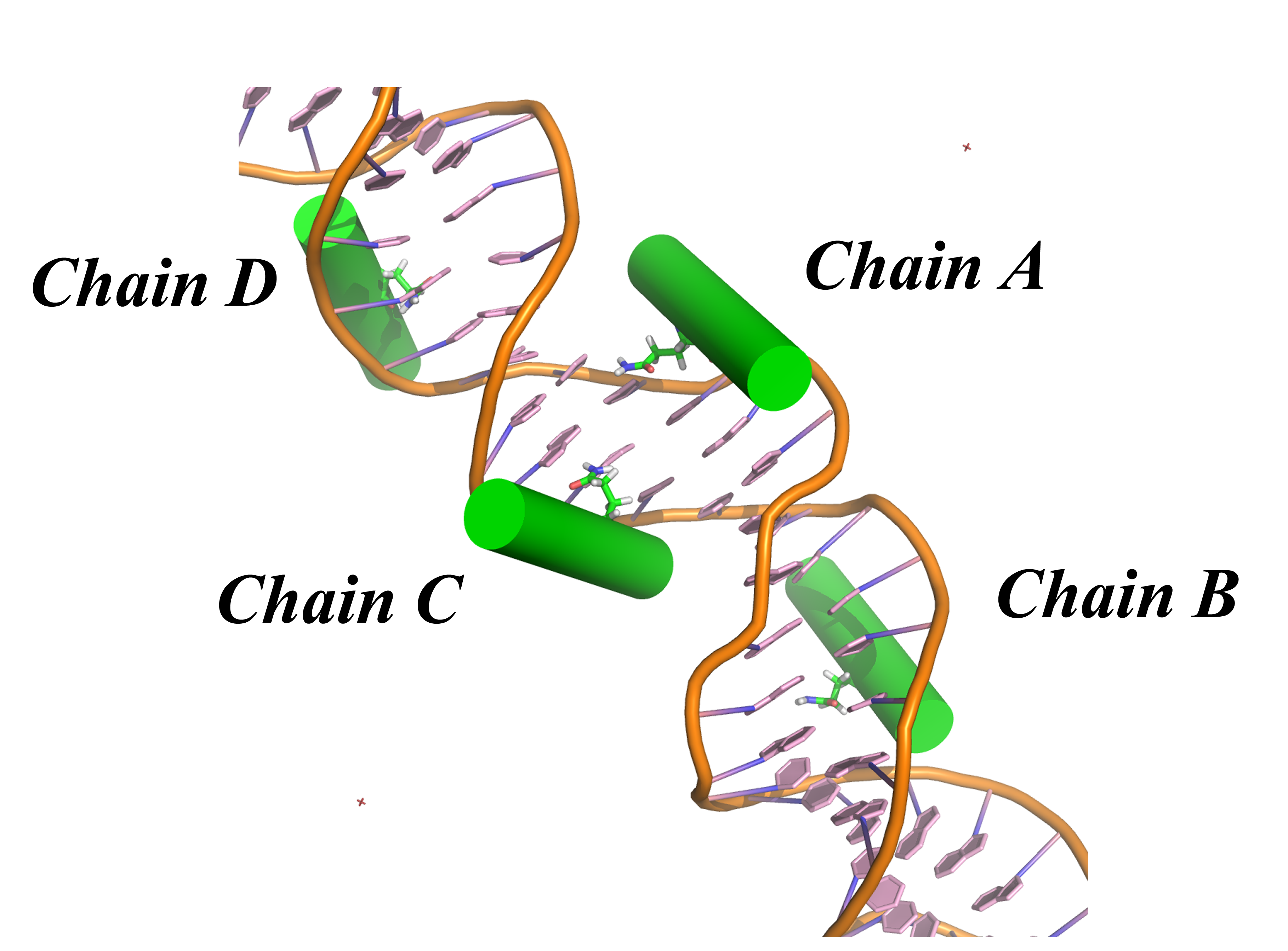

Supplement: S5 Fig — Gln43 is the only residue that interacts with a nucleobase, however not in all subunits. This interaction is guided by the location of Gln43 and base accessibility. Figure represents the side chain orientation of Gln43 in all the subunits and is shown in a stick representation. The DNA binding Helix is shown as green cylinders, while DNA is represented in orange. (TIF) [file pcbi.1004500.s008.tif]

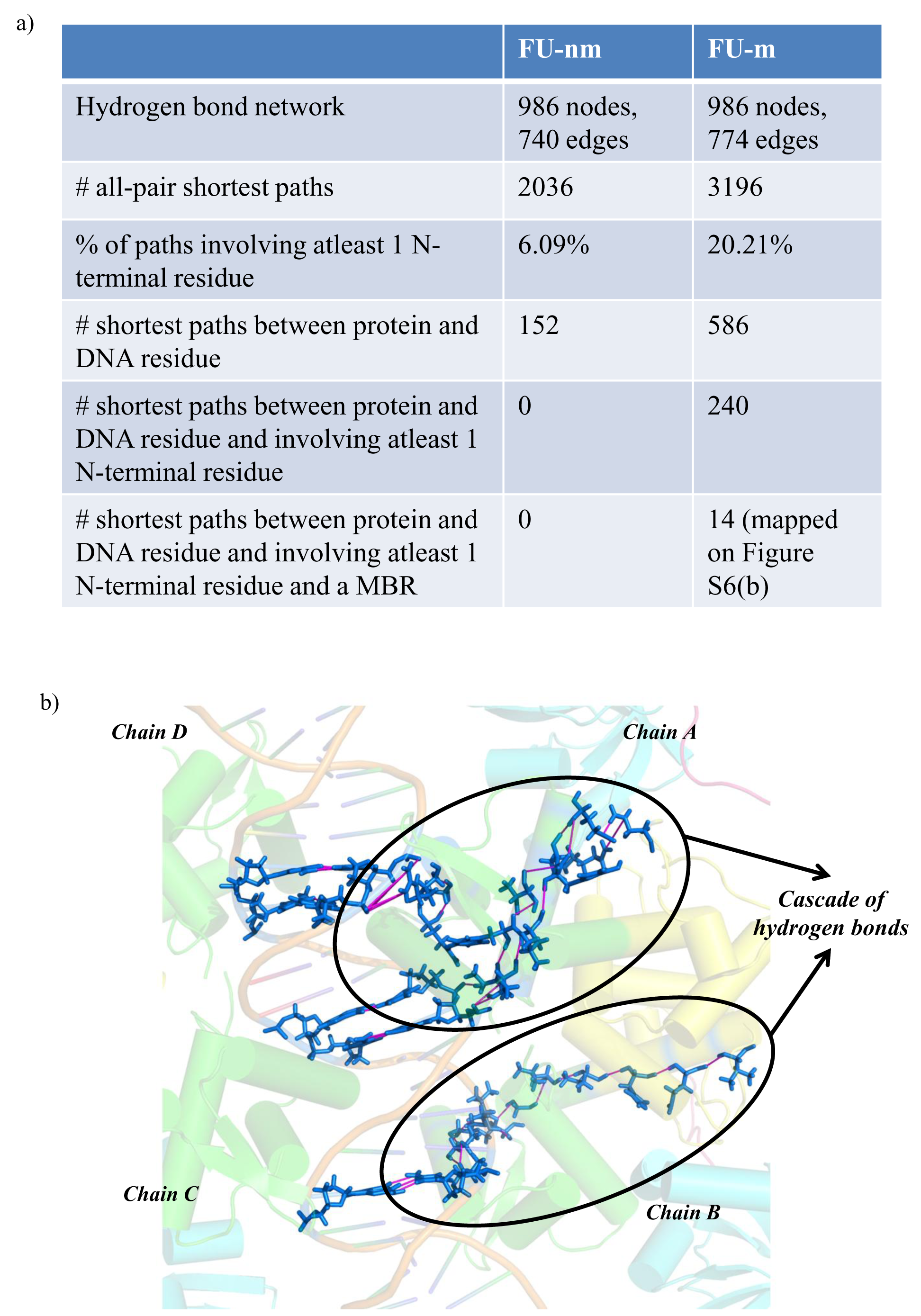

Supplement: S6 Fig — Cascades of hydrogen bonds formed in chain A and B are highlighted. (TIF) [file pcbi.1004500.s009.tif]

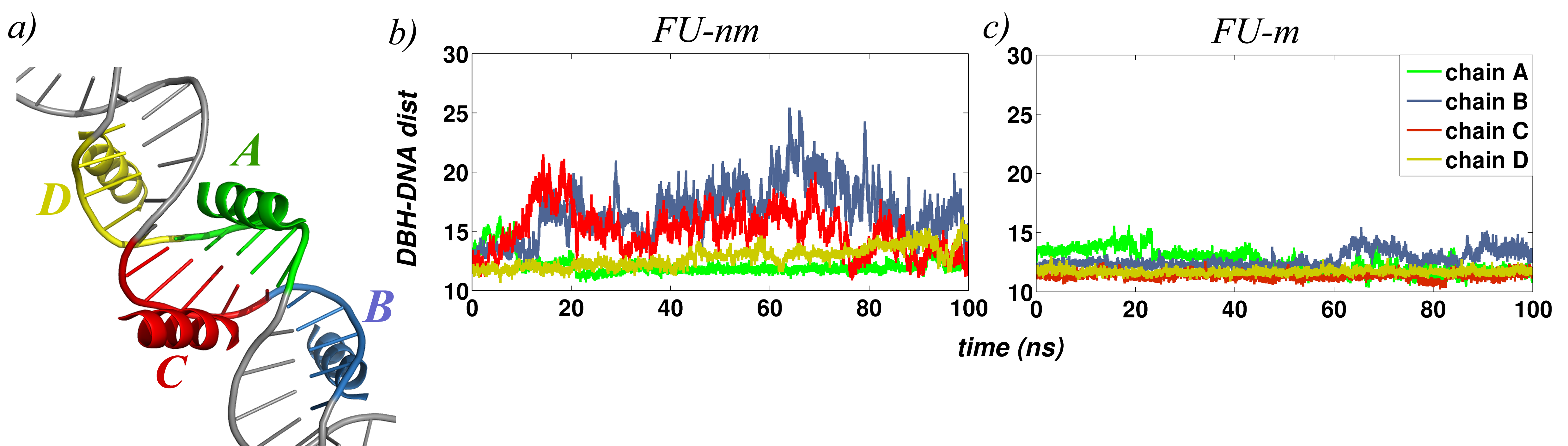

Supplement: S7 Fig — a) DNA binding helices of the different chains and the interacting DNA residues are marked on the structure for representation. The centers of mass of the highlighted residues were used for calculating the DNA-DBH distance in b) FU-nm and c) FU-m. (TIF) [file pcbi.1004500.s010.tif]

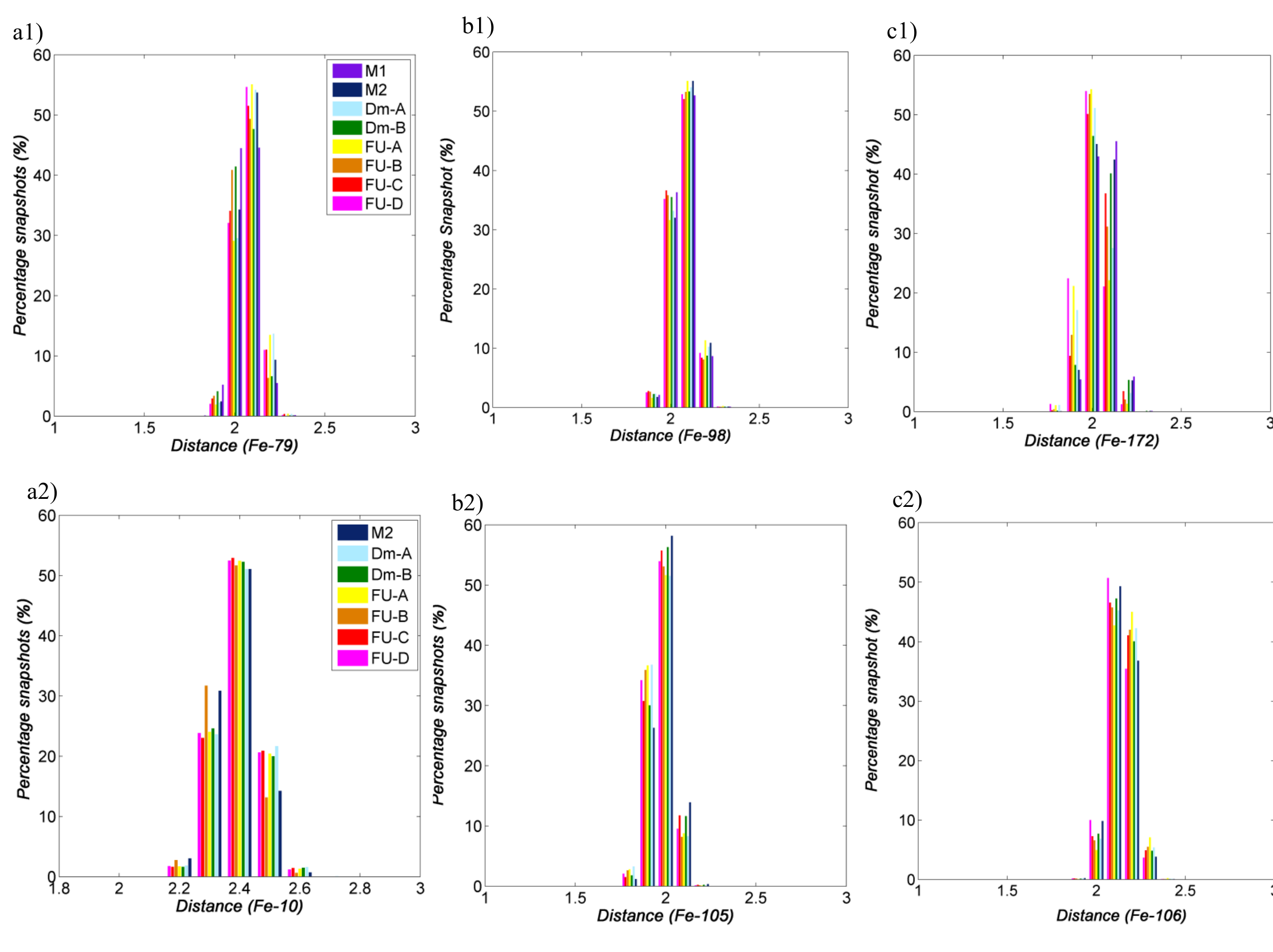

Supplement: S8 Fig — Top panel shows distance values for MS1 and the lower shows values for MS2. X-axis represents the distance values and the y-axis represents the percentage of snapshots with the given distance values. The values match well with experimental results as indicated in Table G of S3 Text. (TIF) [file pcbi.1004500.s011.tif]

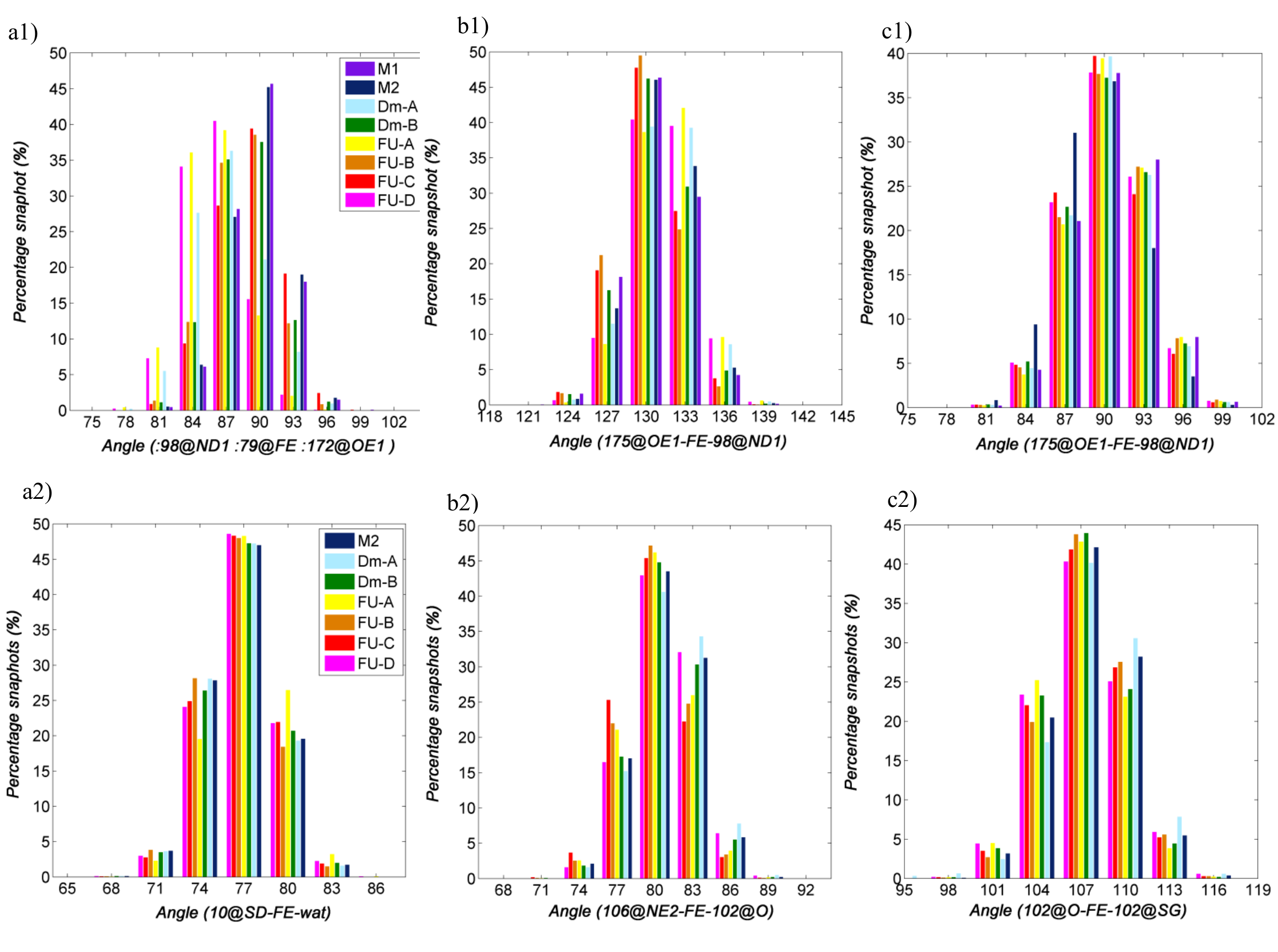

Supplement: S9 Fig — Top panel shows distance values for MS1 and the lower shows values for MS2. X-axis represent the angle values and the y-axis represent the percentage of snapshots with the given values. (TIF) [file pcbi.1004500.s012.tif]
